# Multi-objective resistance-capacitance optimization algorithm: An effective multi-objective algorithm for engineering design problems

**DOI:** 10.1016/j.heliyon.2024.e35921

**Published:** 2024-08-09

**Authors:** Sowmya Ravichandran, Premkumar Manoharan, Deepak Kumar Sinha, Pradeep Jangir, Laith Abualigah, Thamer A.H. Alghamdi

**Affiliations:** a Department of Electrical and Electronics Engineering, Manipal Institute of Technology, Manipal Academy of Higher Education, Manipal, Karnataka, India; b Department of Electrical and Electronics Engineering, Dayananda Sagar College of Engineering, Bengaluru, 560078, Karnataka, India; c Department of Computer Science and Engineering, Faculty of Engineering and Technology, JAIN (Deemed-to-be University), Bangalore, 562112, India; d Department of Biosciences, Saveetha School of Engineering, Saveetha Institute of Medical and Technical Sciences, Chennai, 602105, India; e Computer Science Department, Al al-Bayt University, Mafraq 25113, Jordan; f Artificial Intelligence and Sensing Technologies (AIST) Research Center, University of Tabuk, Tabuk, 71491, Saudi Arabia; g MEU Research Unit, Middle East University, Amman, 11831, Jordan; h Applied Science Research Center, Applied Science Private University, Amman 11931, Jordan; i Centre for Research Impact & Outcome, Chitkara University Institute of Engineering and Technology, Chitkara University, Rajpura, 140401, Punjab, India; j Wolfson Centre for Magnetics, School of Engineering, Cardiff University, Cardiff, CF24 3AA, UK; k Electrical Engineering Department, Faculty of Engineering, Al-Baha University, Al-Baha, 65779, Saudi Arabia; l Jadara University Research Center, Jadara University, Irbid 21110, Jordan

**Keywords:** Engineering design optimization, Honeycomb heat sink design, Multi-objective optimization, Pareto front, Resistance-capacitance optimization algorithm

## Abstract

Focusing on practical engineering applications, this study introduces the Multi-Objective Resistance-Capacitance Optimization Algorithm (MORCOA), a new approach for multi-objective optimization problems. MORCOA uses the transient response behaviour of resistance-capacitance circuits to navigate complex optimization landscapes and identify global optima when faced with many competing objectives. The core approach of MORCOA combines a dynamic elimination-based crowding distance mechanism with non-dominated sorting to generate an ideal and evenly distributed Pareto front. The algorithm's effectiveness is evaluated through a structured, three-phase analysis. Initially, MORCOA is applied to five benchmark problems from the ZDT test suite, with performance assessed using various metrics and compared against state-of-the-art multi-objective optimization techniques. The study then expands to include seven problems from the DTLZ benchmark collection, further validating MORCOA's effectiveness. The final phase involves applying MORCOA to six real-world constrained engineering design problems. Notably, the optimization of a honeycomb heat sink, which is crucial in thermal management systems, is a significant part of this study. This phase uses a range of performance measures to assess MORCOA's practical application and efficiency in engineering design. The results highlight MORCOA's robustness and efficiency in both real-world engineering applications and benchmark problems, demonstrating its superior capabilities compared to existing algorithms. The effective use of MORCOA in real-world engineering design problems indicates its potential as an adaptable and powerful tool for complex multi-objective optimization tasks.

## 1 Introduction

Sophisticated computing methods called Multi-Objective Optimization (MOO) algorithms are made to address problems with numerous, generally conflicting objectives simultaneously. The MOO addresses situations where several criteria need to be taken into account and balanced, in contrast to single-objective optimization, which seeks to identify the optimum solution with respect to a single criterion. These algorithms are essential in a wide range of industries, including engineering, logistics, finance, and healthcare. These help decision-makers negotiate difficult decision-making environments and find the optimal solutions to satisfy a variety of requirements [1,2]. When trade-offs between two or more competing objectives must be determined in real-world circumstances, MOO becomes necessary. For instance, it could be necessary to balance quality and cost in product design, or the objective of environmental management might be to maximize resource use while reducing environmental effects. Due to their inability to take into consideration the complex nature of the decision-making process, traditional optimization techniques that concentrate on a single target are unable to handle these issues sufficiently [3]. MOO techniques are highly influential in a number of domains: (i) With the use of these algorithms, engineers may optimize designs for a variety of factors, including performance, cost, and durability. For example, maximizing a vehicle's design to maximize both safety and fuel efficiency in the automobile industry; (ii) MOO can aid in healthcare logistics by optimizing the distribution of scarce medical resources to maximize benefits across a range of patient groups; (iii) Financial portfolio optimization uses these algorithms to maximize return on investment while minimizing risk, taking into account multiple financial products with varying risk profiles and returns; (iv) They are used to balance economic benefits with environmental protection measures, such as in waste management or water resource planning, where the goal is to maximize waste recycling and minimize disposal costs while ensuring environmental sustainability; (vi) By maximizing supply chain operations across several metrics, including cost, delivery time, and carbon footprint, they help businesses adopt more effective and sustainable supply chain procedures [4,5].

The formation of optimization algorithms to tackle difficult problems has exploded in recent years. The processes seen in nature, such as biological evolution and the behaviour of different creatures, as well as physics and chemistry occurrences and ideas from human cognition, serve as some of the inspirations for these methodologies. These include the Genetic Algorithm (GA) [6], artificial bee colony [7], Particle Swarm Optimization (PSO) [8,9], ant colony optimization [10], bat algorithm [11], Differential Evolutionary (DE) algorithm [12], simulated annealing [13], harmony search [14], and cuckoo search [15], to name a few notable examples. The grey wolf optimizer [16], whale optimizer [17,18], marine predator algorithm [19], slime mould algorithm [20], equilibrium optimizer [21], dragonfly optimizer [22], firefly optimization algorithm [23], reptile search algorithm [24], African vulture optimization algorithm [25], artificial gorilla troop optimizer [26], teaching-learning-based optimization [27], moth flame optimizer [28], political optimizer [29], snake optimizer [30], spotted hyena optimizer [31], mountain gazelle optimizer [32], golden jackal optimizer [33,34], gradient-based optimizer [35], RIME algorithm [36], INFO algorithm [37], butterfly optimization algorithm [38], among other new algorithms, have also been introduced by recent developments. Improved versions of these innovative algorithms are being created to more successfully address engineering difficulties, and they have swiftly emerged as a focus point of academic research. For instance, one creative strategy blended the benefits of swarm algorithm and evolutionary optimization for engineering applications, while another method combined the mechanics of butterfly behaviour with flower pollination processes. The majority of these algorithms were first created as single-objective optimization solutions meant to tackle problems with a single objective in mind [39,40].

However, due to their complexity, real-world issues frequently require solutions that simultaneously balance a number of objectives; these are known as MOO problems (MOOPs). These issues demand specialized algorithms that can negotiate the trade-offs between conflicting objectives. Numerous engineering domains have found themselves in need of the extensive array of MOO algorithms that have been created in recent years. Depending on the viewpoints of the decision-makers concerned, each aim in these challenges usually has varying degrees of relevance [[41], [42], [43]]. The way in which MOO algorithms integrate the decision-maker's choices into the optimization process can be used to categorize them: (i) Priori methods direct the search towards a certain area of the solution space by specifying preferences prior to the optimization process. (ii) Decision-makers can fine-tune their goals and trade-offs by iteratively incorporating preferences into the optimization process through interactive methods. (ii) Posteriori approaches without stating preferences beforehand, generate a set of feasible alternatives. After the optimization process, decision-makers assess these options to determine which is best [44,45].

To address MOOPs, certain strategies have adapted the MOO algorithm into single-objective algorithms through the application of the a priori technique, which involves the integration of weights predetermined by domain experts. However, this approach encounters significant challenges, primarily related to the premature assignment of weights and the consequent difficulty in generating a well-distributed Pareto Front (PF). These hurdles significantly affect the ability of algorithms to produce a PF that encompasses a comprehensive range of optimal solutions [1,46]. In contrast, the a posteriori strategy offers a more robust solution by enabling the identification of a spectrum of Pareto optimal solutions within a single computational effort, obviating the need for advance directives from experts. This method is further categorized into Multi-Objective Evolutionary Algorithm (MOEA) [47], Multi-Objective Cooperative Algorithm (MOCA) [48], and Multi-Objective Swarm Algorithm (MOSA) [49]. A landmark development in this field was the introduction of the Vector Evaluated Genetic Algorithm (VEGA) by Schaffer in 1984, marking the inception of evolutionary algorithms' application to multi-objective contexts [50,51]. Central to the operation of most MOEAs is the principle of Non-Dominated Sorting (NDS), with the Non-Dominated Sorting Genetic Algorithm (NSGA) demonstrating this approach [52]. Its successor, NSGA-II, introduced enhanced NDS processes and mechanisms aimed at diversifying solutions [53]. However, NSGA-II is sometimes evaluated for its tendency towards premature convergence, exposing the loss of high-quality solutions early in the process. The Strength Pareto Evolutionary Algorithm (SPEA) and its subsequent variant, SPEA-II, improved upon the truncation methodology, fitness assignment, and density evaluation strategies of its predecessor to ensure superior convergence and solution distribution [54]. Similarly, the Pareto Envelope-based Selection Algorithm (PESA) and PESA-II emphasize the prioritization of non-dominated solutions, with PESA-II adopting a region-centric selection strategy [55]. Despite SPEA-II's adept search capabilities, its grid structure may impair the uniformity of solution distribution. The MOEA based on Decomposition (MOEA/D) represents another essential development, segmenting MOOPs into discrete subproblems and utilizing an aggregation strategy to leverage insights from adjacent subproblems, thus optimizing the use of computational resources [56]. Nonetheless, this approach might not be as effective for discontinuous PFs, presenting limitations for complex MOOPs. The MOCAs have introduced new methodologies, including parallel processing, hybrid strategies, and coevolution, to enhance optimization processes. A notable instance is the Distributed Cooperative Coevolutionary Algorithm (DCCEA), which advances solution evolution through cooperative subpopulations and an adaptive niche strategy, complemented by a solution archive, to address decision vector segmentation and rule applications [57].

Within the domain of MOSAs, which originate inspiration from natural phenomena and behaviours, Multi-Objective Particle Swarm Optimization (MOPSO) represents a significant adaptation from conventional PSO [58]. MOPSO enhances PSO by integrating multi-objective functions, incorporating mutation strategies, and employing a grid mechanism to improve the distribution of the PF. However, challenges such as suboptimal convergence rates have been noted. Following MOPSO, a variety of innovative MOSAs have been introduced, each drawing on unique aspects of natural and social behaviours to address MOOPs. Among these, the Multi-Objective Grey Wolf Optimizer (MOGWO) stands out for its emulation of grey wolves' social hierarchy and hunting strategies, augmented with an external archive and strategic selection processes for effective MOOP [59,60]. The Multi-Objective Ant Lion Optimizer (MOALO) [61], multi-objective exponential distribution algorithm [62], the Multi-Objective Slime Mould Algorithm (MOSMA) [63], the Multi-Objective Equilibrium Optimizer (MOEO) [64], the Multi-Objective Arithmetic Optimization Algorithm (MOAOA) [65], the Multi-Objective Moth Flame Optimizer [[66], [67], [68]], the Multi-Objective Bat Algorithm (MOBA) [69], the Multi-Objective Cuckoo Search Algorithm (MOCSA) [70], the Multi-Objective Marine Predator Algorithm (MOMPA) [71], etc. are few examples of MOSA and each offering unique strategies to enhance solution quality and PF distribution. Further advancements have seen the development of the MOSA as follows. The Multi-Objective Multi-Verse Optimizer (MOMVO) [72], the Multi-Objective Gradient-Based Optimizer (MOGBO) [73], the Multi-Objective Teaching-Learning-Based Optimizer (MOTLBO) [74,75], the Multi-Objective JAYA (MOJAYA) algorithm [76], the Multi-Objective RAO (MORAO) algorithm [77], are some other MOO algorithms. Each of these algorithms introduces novel mechanisms for navigating the complex landscape of MOPs, with a specific focus on maintaining diversity and achieving broad coverage of the solution space.

Despite these innovations, MOSAs, like their single-objective counterparts, must overcome inherent challenges such as slow convergence rates and the propensity to converge to local optima. The ultimate aim of these algorithms is to furnish decision-makers with a well-optimized PF, addressing the critical parameters of convergence, diversity, and coverage. However, the No Free Lunch (NFL) theorem posits that no single algorithm can universally excel across all MOOPs, highlighting the situational efficacy of these optimizers and underscoring the necessity for versatile, parameter-efficient MOO algorithms capable of addressing a wide array of optimization challenges [78]. This highlights the continuing need for the development of adaptive, efficient MOO algorithms that require minimal parameter tuning to cater to diverse optimization scenarios.

The Resistance-Capacitance Optimization Algorithm (RCOA) draws its unique inspiration from the dynamic response of a Resistance-Capacitance (RC) circuit to abrupt changes in voltage levels [79,80]. This recent mathematical-based algorithm has gathered attention for its initial application in single-objective optimization tasks, demonstrating a promising flexibility for extension into multi-objective contexts. This research paper presents a multi-objective version of RCOA, referred to as MORCOA, which has undergone evaluation through both standard benchmark tests and practical engineering MOOPs. MORCOA not only preserves the foundational characteristics of the original RCOA but also introduces a Dynamic Elimination-Based Crowding Distance (DEBCD) mechanism aimed at enhancing the diversity of solutions [81]. The developments summarized in MORCOA represent significant contributions to the field of optimization, offering a robust tool for addressing complex optimization scenarios that encompass multiple, often competing, objectives. Through the integration of DEBCD along with the NDS method [53], MORCOA stands out for its ability to generate a well-distributed PF, showcasing improved performance in attempting diverse engineering challenges. The primary contributions of this research work can be expressed as follows.

• This research introduces the MORCOA, a new algorithm designed for handling MOOP. MORCOA is based on the transient response behaviour of RC circuits, enabling it to navigate complex optimization landscapes effectively and identify global optima across multiple competing objectives.
• The MORCOA integrates a DEBCD mechanism with NDS to generate an optimal and well-distributed Pareto front. This approach highlights the algorithm's capability by ensuring a balanced exploration and exploitation of the search space.
• The efficacy of MORCOA is rigorously evaluated through a structured three-phase analysis involving benchmark problems from both the ZDT and DTLZ test suites. This thorough evaluation process not only benchmarks MORCOA against leading MOO algorithms but also establishes its effectiveness and efficiency in addressing diverse optimization challenges.
• A significant highlight of this study is the application of MORCOA to the optimization of honeycomb heat sink designs, a critical aspect of thermal management systems. This application demonstrates MORCOA's practical utility in real-world engineering design problems, showcasing its superior performance and adaptability in optimizing the engineering design of honeycomb heat sinks, among other engineering challenges.

The structure of the paper is outlined as follows: Section 2 provides an overview of the Resistance-Capacitance Optimization Algorithm (RCOA). Section 3 details the proposed Multi-Objective Resistance-Capacitance Optimization Algorithm (MORCOA). Section 3 also introduces the basic definitions of MOO algorithms and performance metrics. Section 4 examines the performance of MORCOA across benchmark problems and its application to real-world engineering scenarios, mostly focusing on the design optimization of honeycomb heat sinks. Section 5 concludes the paper and outlines directions for future research.

## 2 Resistance-capacitance optimization algorithm

This section explains the development of the RCOA by leveraging the characteristic transient and steady-state responses of RC circuits to voltage fluctuations [80]. The RCOA algorithm is ingeniously made, drawing parallels between these circuit responses and the optimization phases of exploration and exploitation. The algorithm's foundation lies in the analogy between the transient (temporary) phase and exploration phase and between the steady-state (permanent) phase and exploitation phase. The exploration phase aims to search broadly across the solution space for promising regions, whereas the exploitation phase focuses on refining these solutions to converge towards the optimum. In RCOA, the adaptation of the RC circuit's transient response facilitates exploration, while its steady-state response underpins exploitation. Achieving a balance between these phases is crucial for the algorithm's effectiveness.

The mathematical model of RCOA incorporates the transient response $v_{o}\times e^{-t/\tau }$, to represent exploration and the steady-state response $V_{s}\times \left(1-e^{-t/\tau }\right)$, for exploitation. The progression through time t, which linearly increases from 0 to 1 across iterations, is pivotal in balancing these phases. The expression for time is defined as:

(1) $$t=\frac{it}{Maxit}$$

where it represents the current iteration, Maxit represents the maximum number of iterations, $V_{s}$ denotes the source voltage to the RC circuit, $v_{o}$ denotes the initial voltage of the capacitor, and τ denotes the time constant of the RC circuit. The analogy extends to the capacitor's charging and discharging behaviour in the RC circuit, which is modelled over a period of 5τ when the voltage source is applied or removed. Adjusting the input waveform's time or the RC circuit's time constant alters the capacitor's voltage response, thereby modulating the algorithm's convergence behaviour. The time constant τ is crucial for this modulation and is determined by:

(2) $$\tau =\left(a-b\right)\times r_{1}$$

where a set to a constant value of 5 for optimal performance, and b and $r_{1}$ are random numbers within the range [0,1]. This adjustment allows for the fine-tuning of the algorithm's convergence rate, directly proportional to the time constant τ. The algorithm's implementation simulates the capacitor voltage adjustment by varying the time constant or input frequency, thus establishing a dynamic relationship between voltage (v) and time (t). This principle is vital for RCOA's development, enabling the algorithm to identify and converge on optimal solutions by simulating the RC circuit's behaviour.

The position update mechanism, inspired by the circuit's transient and steady-state responses, is designed to guide the algorithm towards the best solutions. The position vectors for transient-state $\vec{X_{ts}}$ and steady-state $\vec{X_{ss}}$ are updated using the equations:

(3) $$\vec{X_{1}}=\vec{X_{ts}}-\exp\left(-t/\tau \right)$$

(4) $$\vec{X_{2}}=\vec{X_{ss}}\left(1-\exp\left(t/\tau \right)\right)$$

The final position vector is calculated as the sum of these components:

(5) $$\vec{X}\left(t+1\right)=\vec{X_{1}}+\vec{X_{2}}$$

This facilitates a dynamic balance between exploration (when τ<5τ) and exploitation (when τ≥5τ), optimizing the algorithm's performance across different optimization stages. The implementation procedures of RCOA are as follows.

• Define the population size N and maximum iterations Maxit. Generate initial solutions randomly within the bounds of the decision space using Eq. (6).

(6) X=lb+rand×(ub−lb)

• where X denotes the current position of the population, ub denotes the upper bound, lb denotes the lower bound, and rand denotes the uniform random number in the range of 0 and 1.
• Calculate the initial objective function values for the population in the multi-dimensional search space.
• Sort and archive the best objective function values to maintain a record of optimal solutions.
• Implement effective exploration and exploitation strategies to avoid local optima and improve solution quality.
• Adjust the solution positions based on the optimization process, updating the capacitor's voltage (solution values) according to the derived equations.
• If the current iteration equals the maximum allowed iterations, conclude the optimization and return the best solution. Otherwise, repeat from step 2.

The pseudocode of the RCOA is provided in Algorithm 1.

Algorithm 1 Pseudocode for the RCOA

**Table:** 

**Initialize Parameters:** For each individual in the population $X_{i}$, where i=1,2,…,N and N is the size of the population and set initial values for time t and time constant τ.		
**Initial Fitness Evaluation:** Calculate the fitness of each member of the population. Identify transient-state $\vec{X_{ts}}$ and steady-state $\vec{X_{ss}}$ components.		
**While**It<Maxit**do** (current iteration is less than the maximum iterations):		
	**For** each member of the population i:	
		Update the position of the current population member according to Eq. (5).
	**End For**	
	Update the values of t and τ for the next iteration by Eq. (1) and Eq. (2), respectively. Re-evaluate the fitness of each population member.	
	Update the values of $\vec{X_{ts}}$ and $\vec{X_{ss}}$ based on the new positions X(i).	
**End While**		
Return the solution corresponding to the best fitness achieved.

## 3 Proposed multi-objective resistance-capacitance optimization algorithm

This section discusses the basic concepts of the MOO algorithm, non-dominated sorting, and the crowding distance mechanisms. It also discusses the formulation procedure of the proposed MORCOA in detail.

### 3.1 Basic definitions

As suggested by its name, MOO involves optimizing several objectives at the same time. The amount of goals that need to be optimized is typically used to divide this domain into segments. The MOO is a type of optimization that takes into account two or three objectives. It moves into the domain of many-objective optimization, on the other hand, when four or more objectives need to be optimized. The latter is more complicated because it has more aims, which makes it outside the scope of this study but a valuable subject to explore in this realm in the future. The objective is to identify solutions that optimize a collection of objectives simultaneously. Because the objectives frequently conflict with one another, a balance or trade-off must be made between them, which leads to complexity. As opposed to single-objective optimization, which aims to find an optimal solution, this balancing seeks to locate a set of ideal solutions, each of which reflects a unique trade-off between the objectives [82,83]. In mathematical terms, a MOOP can be formulated as follows:

(7) $$\mathrm{Minimize}/Maximize:F\left(\vec{x}\right)={\left(f_{1}\left(\vec{x}\right),f_{2}\left(\vec{x}\right),\ldots ,f_{M}\left(\vec{x}\right)\right)}^{T}$$

Subjectto:

(8) $$\begin{array}{c} g_{i}\left(\vec{x}\right)\geq 0,i=1,2,\ldots ,m\\ h_{j}\left(\vec{x}\right)=0,j=1,2,\ldots ,p\\ \vec{x}={\left(x_{1},x_{2},\ldots ,x_{d}\right)}^{T}\\ {lb}_{i}\leq x_{i}\leq {ub}_{i},i=1,2,\ldots ,d \end{array}\}$$

Where $\vec{x}$ represents the vector of decision variables, $F\left(\vec{x}\right)$ is the vector function of M objectives to be minimized or maximized, d denotes the problem dimensions, $g_{i}\left(\vec{x}\right)$ are inequality constraints, with m being the number of inequality constraints, $h_{j}\left(\vec{x}\right)$ are equality constraints, with p being the number of equality constraints and ${lb}_{i}$ and ${ub}_{i}$ are the lower and upper bounds for the decision variables $x_{i}$. The challenge in MOO is to identify a set of solutions that are non-dominated, meaning no other solution is better for all objectives. These solutions form the PF, illustrating the trade-offs among the objectives that decision-makers must consider. This mathematical framework supports the MOO process, providing a structured approach to navigating the complex landscape of competing objectives.

In the context of MOO, the inherent challenge stems from the diversity of objective functions that represent distinct goals. These objectives, by their very nature, possess unique scales, units, and optimization directions, rendering direct comparisons between solutions within the search space impractical, if not impossible, through conventional relational operators. To address this complexity and facilitate a structured approach to MOO, the concept of Pareto dominance was introduced. This concept serves as a cornerstone for understanding and navigating the multi-dimensional trade-offs between competing objectives. Below, the study investigates definitions of the theory of Pareto dominance and its implications for MOO.

Definition 1 **Pareto Dominance** [82]- Pareto Dominance is a principle used to compare two solutions in the context of multi-objective optimization. A solution A is said to Pareto dominates another solution B if and only if A is no worse than B in all objectives and A is strictly better than B in at least one objective, assuming that all objectives are to be minimized. Mathematically, for two solutions A and B, A dominates B if, for all objectives i, $\left(f_{i}\left(A\right)\leq f_{i}\left(B\right)\right)$, and there exists at least one objective j where $\left(f_{j}\left(A\right)<f_{j}\left(B\right)\right)$, assuming minimization. This concept facilitates the comparison and ranking of solutions based on their performance across multiple criteria without requiring aggregation into a single objective function. Mathematically, the vector $\vec{x}$ dominates the vector $\vec{y}$ if and only if:

(9) $$\forall i\in 1,2,\ldots ,r:f_{i}\left(\vec{x}\right)\leq f_{i}\left(\vec{y}\right)\wedge \exists i\in 1,2,\ldots ,r:f_{i}\left(\vec{x}\right)\leq f_{i}\left(\vec{y}\right)$$

The dominant relationship among $\vec{y}$ and $\vec{x}$ is written as follows: $\vec{x}≻\vec{y}$.

Definition 2 **Pareto Optimality** [82] - A solution is considered Pareto optimal if any other solution in the search space does not dominate it. Pareto optimality implies that no other solution exists that could improve some objectives without worsening at least one other objective. Therefore, a Pareto optimal solution represents a state where an objective cannot be improved without a trade-off in terms of deteriorating performance in another objective, encapsulating the essence of compromise in MOO. A solution $\vec{x}$ is located on the Pareto optimal front if and only if:

(10) $$\neg \exists \vec{y}\in X:\vec{y}≻\vec{x}$$

where X denotes the decision space.

Definition 3 **Pareto Optimal Set** [82] - The Pareto Optimal Set, also known as the Pareto Set, comprises all solutions that are Pareto optimal within the search space. This set embodies the collection of all non-dominated solutions, highlighting the diversity of potential trade-offs among the objectives that are available. Identifying the Pareto Set is a primary goal in multi-objective optimization, as it presents the decision-maker with a spectrum of viable solutions from which to choose based on their preferences and the specific context of the problem. The Pareto set is mathematically expressed as follows.

(11) $$PS=\left\{\vec{x}|\neg \exists \vec{y}\in X:\vec{y}≻\vec{x}\right\}$$

Definition 4 **Pareto Optimal Front** [82] - The Pareto Optimal Front, or PF, represents the mapping of the Pareto Optimal Set in the objective space. It is a graphical depiction that illustrates the trade-offs between the different objectives that the Pareto optimal solutions embody. Each point on the Pareto Front corresponds to a Pareto optimal solution, showcasing the best trade-offs achievable among the objectives. The Pareto Front serves as a valuable tool for decision-makers, providing a visual overview of the possible outcomes and facilitating informed decisions based on the relative importance of each objective. The PF is mathematically expressed as follows.

(12) $$PF=F\left(\vec{x}\right)={\left(f_{1}\left(\vec{x}\right),f_{2}\left(\vec{x}\right),\ldots ,f_{M}\left(\vec{x}\right)\right)}^{T}|\vec{x}\in PS$$

**Fig. 1:**
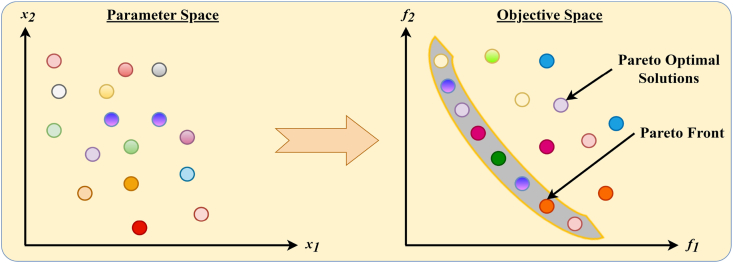
Visual representation of Pareto optimal solutions.

The illustration of the Pareto optimal solution is provided in Fig. 1. Fig. 1 illustrates the concept of Pareto optimal solutions in a multi-objective optimization problem, showing both the parameter space and the objective space. The parameter space represents decision variables, while the objective space plots the objective functions $f_{1}$ and $f_{2}$. Solutions are mapped from the parameter space to the objective space, highlighting the relationship between decision variables and objectives.

The Pareto front is the boundary of non-dominated solutions, representing the best trade-offs between objectives. Dominated solutions lie within the Pareto front, indicating inferior performance, and the dominated solutions are suboptimal because better solutions exist. This visualization clarifies the trade-offs and dominance relationships in multi-objective optimization. Fig. 1 helps to understand the nature of Pareto optimality in MOO, highlighting the trade-offs between competing objectives and the concept of dominance, as well as the relationship between the parameter space and the objective space.

### 3.2 Performance metrics for MOO algorithms

**Fig. 2:**
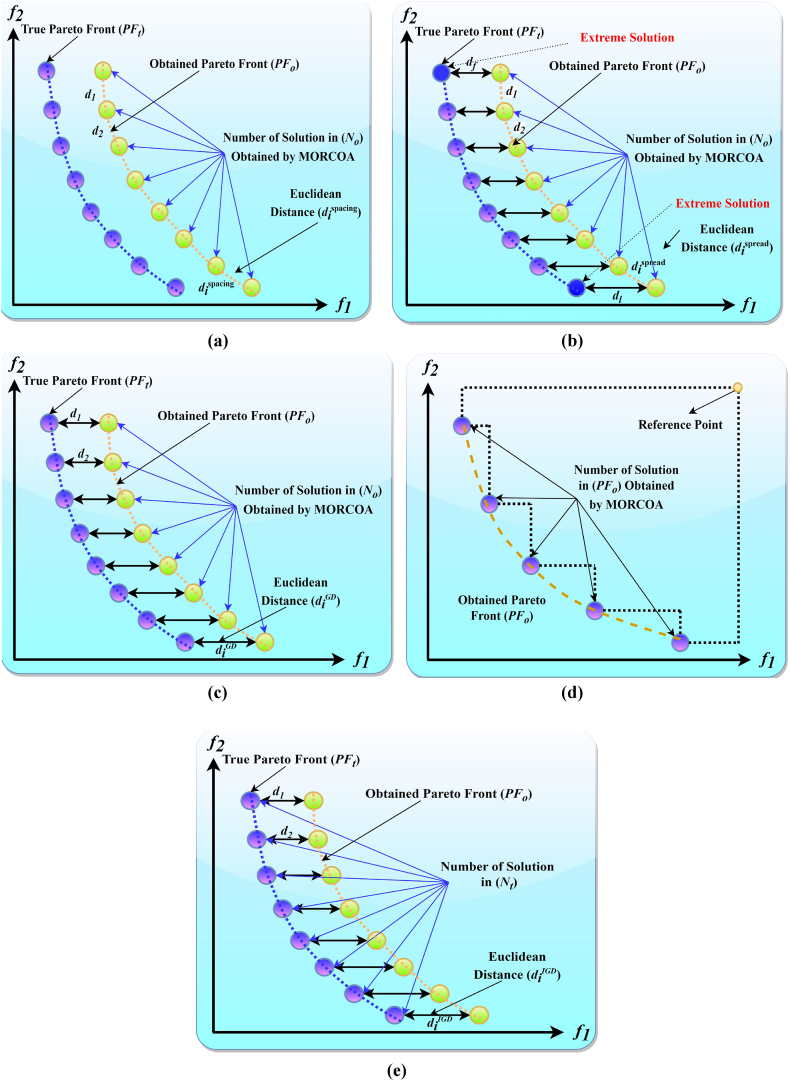
Visual representations of metrics: (a) SP, (b) SD, (c) GD, (d) HV, (e) IGD.

In the context of this study, a meticulous selection of six distinct performance evaluation metrics was employed to assess the outcomes of the research thoroughly. These metrics, namely Spacing (SP), Spread (SD), Generational Distance (GD), Hypervolume (HV), Inverted Generational Distance (IGD), and Run Time (RT), each contribute unique insights into the solution's performance, collectively offering a thorough evaluation of the optimization process's efficacy [[84], [85], [86]]. Fig. 2 illustrates the definitions of all metrics. Collectively, these metrics provide a comprehensive framework for evaluating the algorithm's performance, summarizing its convergence efficiency, solution diversity, and computational feasibility.

Spacing (SP): SP examines the uniformity in the spacing between adjacent solutions along the Pareto front, aiming for a balanced distribution that avoids clustering or excessive sparsity. Smaller SP values denote more uniformly spaced solutions. SP is defined as follows.

(13) $$\text{Spacing}\;\left(SP\right)=\sqrt{\frac{1}{N_{o}-1}\sum_{i=1}^{N_{o}}{\left(\text{mean}\left(d_{i}^{\text{spacing}}\right)-d_{i}^{\text{spacing}}\right)}^{2}}$$

where $N_{o}$ denotes the number of obtained Pareto fronts and $d_{i}^{\text{spacing}}$ denotes the Euclidean distances between consecutive solutions in the objective space.

Spread (SD): SD determines the solutions' distribution range on the PF, evaluating how uniform solutions populate the objective space. A diminished SD value suggests a more evenly distributed set of solutions. The SD metric is calculated as follows.

(14) $$\text{Spread}\;\left(SD\right)=\frac{d_{f}+d_{l}+\sum_{i=1}^{N_{o}-1}\left|d_{i}^{\text{spread}}-\text{mean}\left(d_{i}^{\text{spread}}\right)\right|}{d_{f}+d_{l}+\left(N_{o}-1\right)\cdot \text{mean}\left(d_{i}^{\text{spread}}\right)}$$

where $d_{f}$ denotes the distance between the first solution and the reference point in the objective space, $d_{l}$ denotes the distance between the last solution and the reference point in the objective space and $d_{i}^{\text{spread}}$ is similar to $d_{i}^{\text{spacing}}$ and it denotes the Euclidean distance calculated between consecutive solutions to assess the evenness of the distribution of solutions along the Pareto front.

Generational Distance (GD): GD evaluates the average proximity of solutions generated by the algorithm to the nearest member of the true Pareto front, acting as a convergence indicator. Lower GD values denote solutions that are nearer to the optimal Pareto front. The formula for GD is provided in Eq. (15).

(15) $$\text{Generational}\;\text{Distance}\;\left(\text{GD}\right)=\frac{\sqrt{\sum_{i=1}^{N_{o}}{\left(d_{i}^{\text{GD}}\right)}^{2}}}{N_{o}}$$

(16) $$d_{i}^{\text{GD}}=\min_{j}\left(\sum_{k=1}^{M}\left|f_{k}^{i}\left(\vec{x}\right)-f_{k}^{j}\left(\vec{x}\right)\right|\right)$$

where $d_{i}^{GD}$ is the minimum sum of absolute differences across all M objective functions between solution i and the closest solution j. In other words, it is the distance between a solution in the obtained Pareto front and the closest solution in the true Pareto front.

Hypervolume (HV): HV measures the volume encompassed by the members of a Pareto front within the objective space, serving as a dual indicator of both the convergence and the diversity of solutions within the Pareto front. A superior HV value signifies a Pareto front that spans a broader area, indicative of an enhanced spread and quality of solutions. Nadir points in multi-objective optimization represent the worst objective values found among the Pareto optimal solutions. Specifically, for a Pareto front PF consisting of solutions, the nadir point $s^{nadir}$ is a vector where each component is the maximum value of the corresponding objective across all Pareto optimal solutions. The nadir point $s^{nadir}$ is defined as:

(17) $$s^{nadir}=\left(\max_{s\in PF}f_{1}\left(s\right),\max_{s\in PF}f_{2}\left(s\right),\ldots ,\max_{s\in PF}f_{M}\left(s\right)\right)$$

where $f_{M}\left(s\right)$ is the value of the m-th objective function for solution s in the Pareto front PF.

(18) $$\text{Hyper}\;\text{Volume}\;\left(HV\right)=\Lambda \left(\underset{s\in PF_{o}}{⋃}\left\{s^{\prime }|s⪯s^{\prime }⪯s^{nadir}\right\}\right)$$

where Λ denotes the Lebesgue measure (volume), $PF_{o}$ is the obtained Pareto front, $s⪯s^{\prime }⪯s^{nadir}$ means that $s^{\prime }$ is bounded by the solution s and the nadir point $s^{nadir}$.

Inverted Generational Distance (IGD): IGD, offering a converse perspective to GD, quantifies the average distance from true Pareto front points to the nearest solution identified by the algorithm. It assesses both solution convergence and diversity, with a lower IGD value indicating superior algorithm performance. The IGD computation is as follows.

(19) $$\text{Inverted}\;\text{Generational}\;\text{Distance}\left(IGD\right)=\frac{\sqrt{\sum_{i=1}^{N_{t}}{\left(d_{i}^{\text{IGD}}\right)}^{2}}}{N_{t}}$$

Here, $N_{t}$ represents the count of true Pareto fronts and $d_{i}^{\text{IGD}}$ is the distance between a solution in the true Pareto front and the closest solution in the obtained Pareto front.

Run Time (RT): RT assesses the algorithm's computational efficiency by measuring the time required to derive solutions. In practical applications, where time and computational resources are limited, a shorter RT is indicative of a more efficient algorithm.

### 3.3 Non-dominated sorting

Within the framework of the MORCOA, the procedure for updating solutions adopts a nuanced approach, in contrast to its predecessor, the RCOA, where solution updates are predicated simply on fitness value improvements. Given the multi-objective optimization context, MORCOA incorporates dominance relations to refine its solution update mechanism, which is instrumental in guiding the algorithm toward the true PF. The essence of this refined approach is embedded in the application of NDS, a method that arranges solutions based on a hierarchy of dominance, where solutions less dominated are accorded higher dominance ranks [53]. In MORCOA, solutions undergo an evaluation and update process articulated through NDS, which systematically assigns ranks to solutions reflecting their level of non-dominance. The following elucidates three specific scenarios under which solutions are updated or maintained within the iterative process of MORCOA based on their relative dominance rankings.

• If a candidate solution belonging to the i ^th^ population is ranked on a higher front (F) compared to the current solution's front within the same population, the current solution is supplanted by the candidate solution. For instance, if the candidate solution is positioned on front F2 and the current solution on a lower-ranked front F3, the candidate solution is adopted as the new current solution, reflecting a direct improvement in terms of dominance ranking.
• In instances where both the current and candidate solutions of the i ^th^ population are positioned on the same dominance front, a random selection mechanism with a 50 % probability is employed to decide whether to update the current solution. This scenario accommodates an element of stochasticity, ensuring diversity among solutions of equivalent dominance rank.
• When the dominant front of the candidate solution is ranked lower than that of the current solution for the ith population, the current solution is preserved into the subsequent generation. This decision supports the retention of superior solutions, contributing to a more focused convergence towards the PF.

The solution update strategy articulated in MORCOA, underpinned by NDS, defines a methodical approach to navigating through the complexities of multi-objective optimization. It ensures an effective balance between exploration and exploitation within the search space, thereby enhancing the algorithm's efficacy in multi-objective settings. The mathematical representation of this strategy is given in Eq. (20).

(20) $$x_{i}\left(t+1\right)=\left\{ \begin{array}{c} v_{i}\left(t+1\right),if\;v_{i}\in F_{p},x_{i}\in F_{q}\;\text{and}\;p<q\\ \begin{array}{c} v_{i}\left(t+1\right),\text{if}\;v_{i}\in F_{p},x_{i}\in F_{q},p=q\;\text{and}\;rand<0.5\\ x_{i}\left(t\right),if\;v_{i}\in F_{p},x_{i}\in F_{q},p=q\;\text{and}\;rand\geq 0.5 \end{array}\\ x_{i}\left(t\right){,\mathrm{if}\;v}_{i}\in F_{p},x_{i}\in F_{q}\;\text{and}\;p>q \end{array}\right.$$

where $x_{i}\left(t\right)$ is the position of the i ^th^ individual at iteration t, $x_{i}\left(t+1\right)$ is the updated position of the i ^th^ individual at iteration t+1, $v_{i}\left(t+1\right)$ is the candidate position for the i ^th^ individual at iteration t+1, p and q are indices of the fronts, with p and q being integers, $F_{p}$ signifies the p ^th^ front determined through non-dominated sorting, $F_{q}$ signifies the q ^th^ front determined through non-dominated sorting, and rand is a random number between 0 and 1. This formulation encapsulates the strategic integration of NDS within MORCOA, facilitating a structured and efficient progression towards achieving optimal multi-objective solutions.

### 3.4 Dynamic-elimination-based crowding distance mechanism

Crowding Distance (CD) is a pivotal metric in MOO algorithms, crucial for ensuring uniform solution distribution across the PF [87,88]. CD quantifies the density of solutions surrounding a particular solution within the non-dominated set, playing an essential role in preserving solution diversity during the optimization process. To calculate CD, solutions within the non-dominated set are first organized in ascending order based on their objective function values. The CD for a specific solution, $x_{i}$, is determined by the average distance to its adjacent solutions, $x_{i+1}$ and $x_{i-1}$, effectively measuring the localized solution density. Solutions positioned at the extremities of the objective function value spectrum are assigned an infinite CD to denote their critical role in defining the solution spread. A higher CD value signifies a lower density of neighbouring solutions, indicating a broader diversity among the solutions. In addition to CD, alternative strategies for managing an external archive include the ε-Pareto archiver, grid archiver, and random archiver methods [87]. The grid archiver method segments the objective space into hypercubes and achieves a balanced distribution by selectively pruning solutions within these hypercubes. Conversely, the random archiver method relies on stochastic selection for solution removal from the archive. The ε-Pareto archiver method divides the objective space into ε-defined hypercubes, retaining only the solution closest to the hypercube's origin when multiple non-dominated solutions occupy the same hypercube. The CD is widely regarded as an effective, parameter-independent technique for enhancing solution diversity within MOO algorithms, facilitating the maintenance of a fixed-size external archive. This is accomplished by sequentially removing solutions with the lowest CD to manage the archive's capacity. However, the deletion of a solution necessitates the reevaluation of CDs for the remaining solutions, as the initial removal might alter the subsequent CD ranking, potentially impacting the diversity maintained within the archive.

To mitigate this issue, the implementation of a DEBCD approach is promoted [81]. DEBCD allows for the targeted update of CDs for solutions directly adjacent to the removed solution, thereby minimizing the need for widespread recalculations. Specifically, upon the removal of a solution with the smallest CD, the CDs of the immediately preceding and succeeding solutions are recalculated to reflect the new configuration. This approach ensures that only the CDs of solutions in close proximity to the removed solution are adjusted, enhancing the efficiency of the archive management process. The adoption of DEBCD significantly streamlines the process of maintaining diversity within the PS when constructing an external archive. By necessitating the recalculation of CDs for a limited number of solutions directly impacted by the removal, DEBCD optimizes the computational demands of this process, which is particularly beneficial in scenarios involving the removal of multiple solutions for archive management. This methodological refinement underscores the dynamic and computational efficiency of employing DEBCD in MOO algorithms, particularly in ensuring an even and representative distribution of solutions across the PF.

Before the elimination of the solution with the lowest CD, identified as the i ^th^ solution, the CDs for the solutions immediately adjacent to it are calculated using the following formulas:

For the preceding solution:

(21) $$d_{k}\left(x_{i-1}\right)=\frac{\left|f_{k}\left(x_{i}\right)-f_{k}\left(x_{i-2}\right)\right|}{\max\left(f_{k}\right)-\min\left(f_{k}\right)},D\left(x_{i-1}\right)=\sum_{k=1}^{m}d_{k}\left(x_{i-1}\right)$$

For the succeeding solution:

(22) $$d_{k}\left(x_{i+1}\right)=\frac{\left|f_{k}\left(x_{i}\right)-f_{k}\left(x_{i+2}\right)\right|}{\max\left(f_{k}\right)-\min\left(f_{k}\right)},D\left(x_{i+1}\right)=\sum_{k=1}^{m}d_{k}\left(x_{i+1}\right)$$

where $x_{i}$ denotes the i ^th^ solution in the current population, $f_{k}\left(x\right)$ denotes the value of the k ^th^ objective function for solution x, $d_{k}\left(x_{i-1}\right)$ denote the individual distance measurement based on the k ^th^ objective for the solution immediately preceding $x_{i}$, $d_{k}\left(x_{i+1}\right)$ denotes the individual distance measurement based on the k ^th^ objective for the solution immediately succeeding $x_{i}$, $D\left(x_{i-1}\right)$ denotes the aggregate crowding distance for the solution immediately preceding $x_{i}$, $D\left(x_{i+1}\right)$ denotes the aggregate crowding distance for the solution immediately succeeding $x_{i}$, $\max\left(f_{k}\right)$ denotes the maximum value of the k ^th^ objective function in the current population, $\min\left(f_{k}\right)$ denotes the minimum value of the k ^th^ objective function in the current population, and m denotes the total number of objective functions. Following the removal of the i ^th^ solution due to its minimal CD, the CDs for the adjacent solutions, specifically the one preceding and the one succeeding the i ^th^ solution, are recalculated and updated as follows:

For recalculating the CD of the preceding solution:

(23) $$d_{k}\left(x_{i-1}\right)=\frac{\left|f_{k}\left(x_{i+1}\right)-f_{k}\left(x_{i-2}\right)\right|}{\max\left(f_{k}\right)-\min\left(f_{k}\right)},D\left(x_{i-1}\right)=\sum_{k=1}^{m}d_{k}\left(x_{i-1}\right)$$

For recalculating the CD of the succeeding solution:

(24) $$d_{k}\left(x_{i+1}\right)=\frac{\left|f_{k}\left(x_{i-1}\right)-f_{k}\left(x_{i+2}\right)\right|}{\max\left(f_{k}\right)-\min\left(f_{k}\right)},D\left(x_{i+1}\right)=\sum_{k=1}^{m}d_{k}\left(x_{i+1}\right)$$

**Fig. 3:**
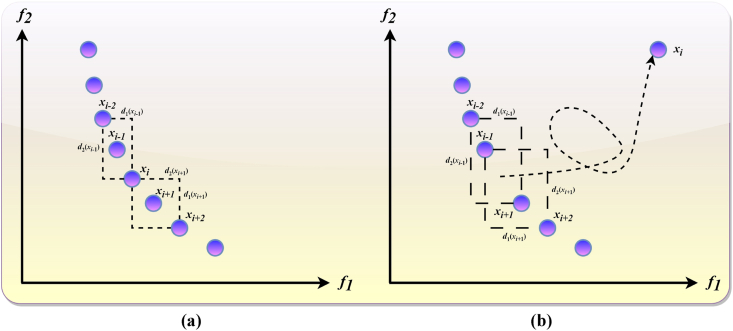
Modification of CD of solutions nearer to removed one using dynamic-elimination CD for two objectives. (a) CDs of $x_{i-1}$ and $x_{i+1}$ before $x_{i}$ is removed, (b) CDs of $x_{i-1}$ and $x_{i+1}$ after $x_{i}$ is removed.

The DEBCD modifies only the CDs of solutions that are directly adjacent to the removed solution, thus minimizing the need for extensive CD recalculations across the entire solution set. This method is particularly efficient as it requires recalculating the CDs for only 2n solutions when n solutions are designated for removal to maintain the external archive, significantly optimizing the computational workload. This approach, highlighted in the visualization provided in Fig. 3, showcases the practical application of DEBCD in scenarios involving two objectives, demonstrating how CDs are dynamically adjusted to maintain an evenly distributed and diverse set of solutions within the PS, thereby enhancing the efficiency of MOO algorithms.

The pseudocode outlines the procedure for managing an external archive using the DEBCD method shown in Algorithm 2, ensuring that the archive remains within a predetermined size while maintaining solution diversity through dynamic CD updates.

Algorithm 2 DEBCD Method for External Archive Maintenance

**Table:** 

***n:*** Total number of solutions in the fixed external archive. **p:** Number of solutions to be removed to maintain the fixed external archive size.		
	**For** each target solution j from 1 to p:	
		Sort the solutions by their CD for i in the range (2,3,…,n−1). Update the CD of the (i−1)^th^ solution using Eq. (23). Update the CD of the (i+1)^th^ solution using Eq. (24).
	**End For**

Within the domain of optimizing solution diversity in MOO, various strategies have been explored to enhance the distribution of solutions across the Pareto Front. Among these, the authors in Ref. [89] proposed the Crowding Distance Elimination (CDE) method, specifically designed to improve solution diversity. Concurrently, another set of researchers in Ref. [88] introduced the Dynamic CD-based Diversity Maintenance Approach (DCD-DMA), with a particular focus on expanding solution diversity more uniformly across the objective space. A key feature shared by CDE, DEBCD, and DCD-DMA is their strategic focus on solutions exhibiting the minimum CD. The removal of such solutions triggers a re-evaluation and potentially a reordering of the remaining solutions based on their CD. The primary difference among these methodologies lies in their approach to recalibrating CDs post-removal of a solution. While CDE and DCD-DMA necessitate a comprehensive recalculation of CDs for all remaining solutions in the solution set, DEBCD distinguishes itself by recalibrating CDs exclusively for those solutions directly adjacent to the removed solution. This nuanced recalibration strategy employed by DEBCD markedly reduces the computational demands associated with recalculating crowding distances, transitioning from the complexity of $O\left(TMN^{2}\right)$ to O(TMN). Here, T represents the total number of generations, M denotes the number of objectives being optimized, and N signifies the population size. By focusing only on adjacent solutions for CD recalibration, DEBCD not only minimizes computational overhead but also enhances the method's efficiency, particularly in scenarios involving extensive solution sets. This efficiency proves to be especially advantageous in maintaining diversity within large solution sets, streamlining the dynamic management of solution distribution across the PF. By adopting DEBCD, practitioners can ensure a more computationally efficient and effective approach to preserving solution diversity in MOOPs, thereby facilitating a more balanced exploration and exploitation of the search space.

### 3.5 Formulation of the proposed algorithm

The methodology of MOO initiates with the creation of an initial population. During this phase, the values of the objective functions are evaluated, and solutions that are non-dominated according to NDS criteria are identified. Following this, the equations governing the RCOA, specifically Eq. (1) through 6, are applied to derive a new set of population members. The selection process for candidates advancing to the subsequent generation involves an analytical comparison of their performance across both previous and current evaluations. In scenarios involving two distinct populations labelled $P_{1}$ and $P_{2}$, the determination of dominance is conducted through a comparative analysis of their respective objective function outcomes, denoted as $f_{1}$ and $f_{2}$. The dominance of $P_{1}$ over $P_{2}$ is established if every component of $f_{1}$ is either equivalent to or better than (lower in cases where minimization is the goal) its corresponding component in $f_{2}$, with at least one element of $f_{1}$ being strictly superior (lower). Solutions that any other candidate does not outperform are classified as NDS solutions and are accordingly assigned a primary rank. Subsequent to the extraction of these top-ranked non-dominated solutions, the process iteratively continues to unveil the next tiers of non-dominated solutions, assigning them increasing ranks until every agent within the population is ranked.

**Fig. 4:**
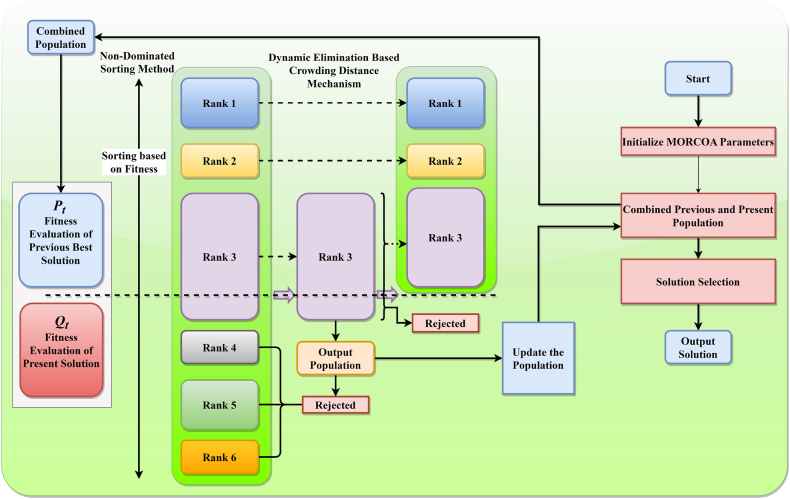
Procedure of NDS method.

**Fig. 5:**
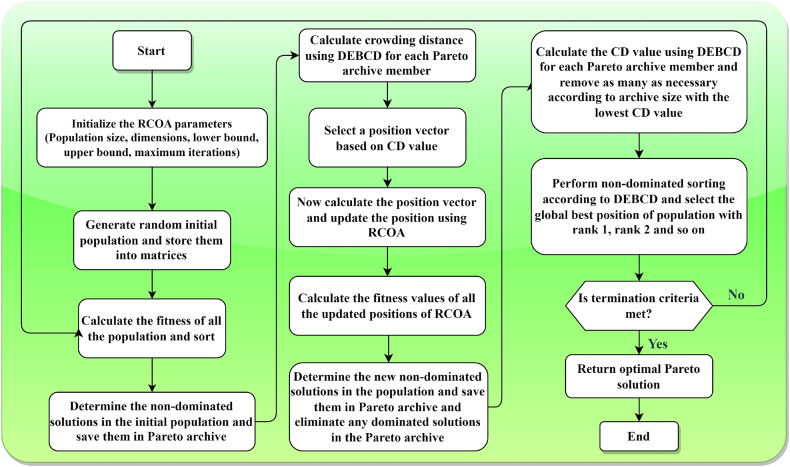
Flowchart of suggested MORCOA.

The integration and ranking of both existing and newly identified agents are based on their dominance ranks, prioritizing those with lesser degrees of domination. The selection procedure for the ensuing generation commences by accumulating the highest-ranked non-dominated solutions into a new, initially empty collection. Should the aggregate of agents within this collection exceed the designated population limit, the DEBCD method is employed to restrict the agent selectively count within the new collection. The DEBCD strategy is instrumental in managing the set of NDS solutions to adhere to the specified population quota while simultaneously promoting diversity, characterized by a broad and uniform distribution of solutions. Consequently, n solutions exhibiting the most significant CD values are retained for inclusion in the next generation. In instances where the count of rank-1 NDS solutions falls short of the population threshold, solutions with rank-2 designation are incorporated into the collection, a process that is iteratively repeated until the newly formed set aligns with the population size criteria. This NDS-centric selection mechanism is visually represented in Fig. 4, for clarity. Further, **Algorithm 3** delineates the pseudocode for implementing the MORCOA strategy. The procedure for MORCOA is initiated with predetermined parameters such as termination criteria Maxit and population size N. An initial random population $P_{0}$ within feasible limits is established and subjected to assessment. Subsequently, DEBCD and NDS methodologies are applied to $P_{0}$, leading to the formation of a new population $P_{j}$, which is then amalgamated with $P_{0}$ to generate population $P_{i}$. This combined population $P_{i}$ is organized based on DEBCD and NDS rankings, from which the optimal solutions are selected to promote a new parent population. This cyclical process is reiterated until the specified termination criteria are satisfied, ensuring a systematic and efficient progression towards the optimization objectives. The operational flow of MORCOA is illustrated in Fig. 5 to provide a comprehensive understanding of the algorithm's implementation.

Algorithm 3 Execution Procedure for MORCOA

**Table:** 

Initialize RCOA parameters and the initial population $P_{0}\left(t=0\right)$ within the feasible solution space S.	
	1. Evaluate the objective function values F for the initially generated population $P_{0}$. 2. Apply NDS to $P_{0}$ to determine non-dominated ranks and identify distinct fronts. 3. For each identified front, calculate CD values using Eqs. (23), (24)). 4. Utilize the RCOA as outlined in **Algorithm 1** to update the solutions, resulting in population $P_{j}$. 5. Merge the initial population $P_{0}$ with the updated population $P_{j}$ to form a combined population $P_{i}=P_{0}\cup P_{j}$. 6. Reassess the combined population $P_{i}$ within the objective space F. 7. Organize $P_{i}$ according to non-dominated rankings and associated CD values. 8. Update the initial population $P_{0}$ by selecting N top-ranked members from $P_{i}$ based on their non-dominated rank and CD.
**Return**: Updated population representing the best optimal fronts.

### 3.6 Time and space complexity of MORCOA

#### 3.6.1 Time complexity

Initializing the population $P_{0}$ and setting RCOA parameters takes O(N×dim) time. Evaluating the objective function values for the initial population $P_{0}$ requires O(N×dim) time. Applying NDS to $P_{0}$ to determine non-dominated ranks and identify distinct fronts takes $O\left(M\times N^{2}\right)$ time, where \ M is the number of objectives. Calculating the crowding distance values for each identified front using DEBCD takes $O\left(N^{2}\right)$ time. Utilizing RCOA to update the solutions, resulting in population $P_{j}$, takes O(N×dim) time per iteration. For Maxit iterations, this takes O(N×dim×Maxit) time. Merging the initial population $P_{0}$ with the updated population $P_{j}$ to form a combined population $P_{i}$ takes O(N) time. Reassessing the combined population $P_{i}$ within the objective space F requires O(N×dim) time. Re-applying NDS on $P_{i}$ takes $O\left(M\times N^{2}\right)$ time. Selecting N top-ranked members from $P_{i}$ based on their non-dominated rank, and CD takes O(NlogN) time. Considering all steps, the overall time complexity of MORCOA is dominated by the non-dominated sorting and RCOA updates. Hence, the overall time complexity is $O\left(\text{Maxit}\times N\times \dim\right)+O\left(M\times N^{2}\right)+O\left(N^{2}\right)$ and simplified as $O\left(\text{Maxit}\times N\times \dim+M\times N^{2}\right)$.

#### 3.6.2 Space complexity

Storing the population $P_{0}$ requires O(N×dim) space. Storing the objective function values for each individual in the population requires O(N×M) space. Storing crowding distance values and non-dominated ranks for each individual requires O(N) space. The combined population $P_{i}$ and its reassessment also requires O(N×dim) space. The storage of the population and objective function values dominates the overall space complexity of MORCOA. Therefore, the overall space complexity is O(N×dim)+O(N×M). Simplifying, the overall space complexity can be expressed as O(N×(dim+M)).

### 3.7 Best Compromise Solutions

The Technique for Order Preference by Similarity to Ideal Solution (TOPSIS) is a multi-criteria decision-making method that identifies solutions that have the shortest distance from the positive ideal solution (PIS) and the farthest distance from the negative ideal solution (NIS) [90,91]. When applied to the MORCOA, TOPSIS can be utilized to determine the Best Compromise Solutions (BCS) among the set of Pareto optimal solutions generated by MORCOA. This approach effectively balances between multiple conflicting objectives by selecting solutions that exhibit an optimal compromise. The steps to implement TOPSIS for MORCOA are as follows.

Step 1 Normalization of the Decision Matrix - The first step involves normalizing the decision matrix to eliminate the effects of differing units and scales among the objectives. The normalized value $r_{ij}$ is calculated using Eq. (25).

(25) $$r_{ij}=\left\{ \begin{array}{c} \frac{x_{ij}}{\sqrt{\sum_{k=1}^{n}x_{kj}^{2}}},if\;\sum_{k=1}^{n}x_{kj}^{2}\neq 0\\ 0,if\;\sum_{k=1}^{n}x_{kj}^{2}=0 \end{array}\right.$$

where $x_{ij}$ is the value of the $j^{th}$ criterion for the $i^{th}$ alternative, and n is the number of alternatives.

Step 2 **: Determination of the PIS and NIS -** The PIS $A^{+}$ is the solution with the best value for each criterion among the set of Pareto optimal solutions, and the NIS $A^{-}$ is the solution with the worst value for each criterion. They are defined as follows.

(26) $$\begin{array}{c} A^{+}=\left\{\max\;r_{ij}|j\in J\right\},\text{for}\;\text{beneficial}\;\text{criteria}\\ A^{-}=\left\{\min\;r_{ij}|j\in J\right\},\text{for}\;\text{non}-\text{beneficial}\;\text{criteria} \end{array}\}$$

Step 3 **Calculation of the Distance to PIS and NIS -** The next step is to calculate the distance of each Pareto optimal solution from the PIS and NIS. The distance from the PIS $D_{i}^{+}$ and the distance from the NIS $D_{i}^{-}$ for each solution i are computed as follows.

(27) $$\begin{array}{c} D_{i}^{+}=\sqrt{\sum_{j=1}^{m}{\left(r_{ij}-r_{j}^{+}\right)}^{2}}\\ D_{i}^{-}=\sqrt{\sum_{j=1}^{m}{\left(r_{ij}-r_{j}^{-}\right)}^{2}} \end{array}\}$$

where m is the number of criteria, $r_{j}^{+}$ and $r_{j}^{-}$ are the values of criterion j for the PIS and NIS, respectively.

Step 4 **Calculation of the Relative Closeness to the Ideal Solution -** The relative closeness of each solution to the ideal solution is calculated to identify the best compromise solution. The relative closeness $C_{i}$ of solution i is given by:

(28) $$C_{i}=\left\{ \begin{array}{c} \frac{D_{i}^{-}}{D_{i}^{+}+D_{i}^{-}},if\;D_{i}^{+}+D_{i}^{-}\neq 0\\ 0,if\;D_{i}^{+}+D_{i}^{-}=0 \end{array}\right.$$

Solutions with higher $C_{i}$ values are considered better, as they are closer to the PIS and further from the NIS.

Step 5 **Selection of the BCS -** The solution(s) with the highest relative closeness to the ideal solution $C_{i}$ is selected as the best compromise solution.

By implementing TOPSIS in MORCOA, decision-makers can systematically evaluate and select the most suitable compromise solution from the set of Pareto optimal solutions, effectively addressing the trade-offs between multiple objectives. This method provides a clear, mathematical framework for navigating the complex decision space characteristic of multi-objective optimization problems.

## 4 Results and discussions

This section explains the research findings and the experimental analyses undertaken to evaluate the MORCOA's efficacy and demonstrate its capabilities. To ensure the robustness and statistical validation of the outcomes, each experimental run was independently conducted 30 times, with the experiments configured to a population size of 40 and a cap of 1000 iterations [92]. The computational work was carried out on MATLAB R2023b, hosted on a system powered by an Intel Core i5 processor, clocked at 2.5 GHz, and supported by 16 GB of RAM. The assessment of MORCOA's performance was systematically approached through the employment of three distinct categories of benchmark test functions.

(i) Zitzler–Deb–Thiele (ZDT) Benchmark Suite [41]: This includes a series of test functions, specifically ZDT1 through ZDT4 and ZDT6. These functions are detailed in Table 1 and are characterized by having dual objective functions, mirroring the dual-objective nature prevalent in Pareto optimization, especially within engineering domains.

**Table 1:** List of ZDT benchmark test functions.

Name	Objective Functions and Constraints	*dim*	Bounds	Features
ZDT1	$f_{1}\left(x\right)=x_{1}$	30	$x_{i}\in \left[0,1\right]$, i=2,…,dim	Convex
	$f_{2}\left(x,g\right)=g\left(x\right).\left(1-\sqrt{\frac{f_{1}}{g\left(x\right)}}\right)$			
	$g\left(x\right)=1+\frac{9}{n-1}.\sum_{i=2}^{n}x_{i}$			
ZDT2	$f_{1}\left(x\right)=x_{1}$	30	$x_{i}\in \left[0,1\right]$, i=2,…,dim	Non-convex
	$f_{2}\left(x,g\right)=g\left(x\right).\left(1-{\left(\sqrt{\frac{f_{1}}{g\left(x\right)}}\right)}^{2}\right)$			
	$g\left(x\right)=1+\frac{9}{n-1}.\sum_{i=2}^{n}x_{i}$			
ZDT3	$f_{1}\left(x\right)=x_{1}$	30	$x_{i}\in \left[0,1\right]$, i=2,…,dim	Convex, disconnected
	$f_{2}\left(x,g\right)=g\left(x\right).\left(1-\sqrt{\frac{f_{1}}{g\left(x\right)}}-\frac{f_{1}}{g\left(x\right)}.\sin\left(10\pi f_{1}\right)\right)$			
	$g\left(x\right)=1+\frac{9}{n-1}.\sum_{i=2}^{n}x_{i}$			
ZDT4	$f_{1}\left(x\right)=x_{1}$	10	$x_{i}\in \left[0,1\right]$, i=2,…,dim	Non-convex
	$f_{2}\left(x,g\right)=g\left(x\right).\left(1-\sqrt{\frac{f_{1}}{g\left(x\right)}}\right)$			
	$g\left(x\right)=1+10.\left(n-1\right)+\sum_{i=2}^{n}\left(x_{i}^{2}-10\text{co}s\left(4\pi x_{i}\right)\right)$			
ZDT6	$f_{1}\left(x\right)=1-\exp\left(4x_{1}\right).\sin^{6}\left(6\pi x_{1}\right)$	10	$x_{i}\in \left[0,1\right]$, i=2,…,dim	Non-convex, Nonuniformly spaced
	$f_{2}\left(x,g\right)=g\left(x\right).\left(1-{\left(\frac{f_{1}}{g\left(x\right)}\right)}^{2}\right)$			
	$g\left(x\right)=1+\frac{9}{n-1}.{\left[\frac{\sum_{i=2}^{n}x_{i}}{9}\right]}^{0.25}$
(ii) Deb–Thiele–Laumanns–Zitzler (DTLZ) Benchmark Functions [93]: Distinguished by their scalability, the DTLZ suite permits the extension to an arbitrary number of objectives, rendering it an indispensable asset for exploring challenges in "many-objective" optimization. These functions are detailed in Table 2.

**Table 2:** Lists of three objective DTLZ benchmark test functions.

Name	Objective Functions and Constraints	*dim*	Bounds	Features
DTLZ1	$f_{1}\left(x\right)=\frac{1}{2}x_{1}x_{2}\left(1+g\left(x\right)\right)$	12	[0,1]	Triangular-linear
	$f_{2}\left(x\right)=\frac{1}{2}x_{1}\left(1-x_{2}\right)\left(1+g\left(x\right)\right)$			
	$f_{3}\left(x\right)=\frac{1}{2}\left(1-x_{1}\right)\left(1+g\left(x\right)\right)$			
	$g\left(x\right)=100\left[10+\sum_{i=3}^{n}{\left(x_{i}-0.5\right)}^{2}-\text{co}s\left(20\pi \left(x_{i}-0.5\right)\right)\right]$			
DTLZ2	$f_{1}\left(x\right)=\cos\left(\frac{\pi }{2}x_{1}\right)\cos\left(\frac{\pi }{2}x_{2}\right)\left(1+g\left(x\right)\right)$	12	[0,1]	Inverted-concave
	$f_{2}\left(x\right)=\cos\left(\frac{\pi }{2}x_{1}\right)\sin\left(\frac{\pi }{2}x_{2}\right)\left(1+g\left(x\right)\right)$			
	$f_{3}\left(x\right)=\sin\left(\frac{\pi }{2}x_{1}\right)\left(1+g\left(x\right)\right)$			
	$g\left(x\right)=\sum_{i=3}^{n}{\left(x_{i}-0.5\right)}^{2}$			
DTLZ3	$f_{1}\left(x\right)=\cos\left(\frac{\pi }{2}x_{1}\right)\cos\left(\frac{\pi }{2}x_{2}\right)\left(1+g\left(x\right)\right)$	12	[0,1]	Inverted-concave
	$f_{2}\left(x\right)=\cos\left(\frac{\pi }{2}x_{1}\right)\sin\left(\frac{\pi }{2}x_{2}\right)\left(1+g\left(x\right)\right)$			
	$f_{3}\left(x\right)=\sin\left(\frac{\pi }{2}x_{1}\right)\left(1+g\left(x\right)\right)$			
	$g\left(x\right)=100\left[10+\sum_{i=3}^{n}{\left(x_{i}-0.5\right)}^{2}-\text{co}s\left(20\pi \left(x_{i}-0.5\right)\right)\right]$			
DTLZ4	$f_{1}\left(x\right)=\cos\left(\frac{\pi }{2}x_{1}^{\alpha }\right)\cos\left(\frac{\pi }{2}x_{2}^{\alpha }\right)\left(1+g\left(x\right)\right)$	12	[0,1]	Inverted-concave
	$f_{2}\left(x\right)=\cos\left(\frac{\pi }{2}x_{1}^{\alpha }\right)\sin\left(\frac{\pi }{2}x_{2}^{\alpha }\right)\left(1+g\left(x\right)\right)$			
	$f_{3}\left(x\right)=\sin\left(\frac{\pi }{2}x_{1}^{\alpha }\right)\left(1+g\left(x\right)\right)$			
	$g\left(x\right)=\sum_{i=3}^{n}{\left(x_{i}-0.5\right)}^{2}$			
DTLZ5	$f_{1}\left(x\right)=\cos\left(\frac{\pi }{2}\theta_{1}\right)\cos\left(\frac{\pi }{2}\theta_{2}\right)\left(1+g\left(x\right)\right)$	12	[0,1]	Inverted-Curved
	$f_{2}\left(x\right)=\cos\left(\frac{\pi }{2}\theta_{1}\right)\sin\left(\frac{\pi }{2}\theta_{2}\right)\left(1+g\left(x\right)\right)$			
	$f_{3}\left(x\right)=\sin\left(\frac{\pi }{2}\theta_{1}\right)\left(1+g\left(x\right)\right)$			
	$\theta_{1}=x_{1}.\left(\frac{\pi }{2}\right),\theta_{2}=\frac{\pi }{4.\left(1+g\left(x\right)\right)}.\left(1+2x_{2}.g\left(x\right)\right)$$g\left(x\right)=\sum_{i=3}^{n}{\left(x_{i}-0.5\right)}^{2}$			
DTLZ6	$f_{1}\left(x\right)=\cos\left(\frac{\pi }{2}\theta_{1}\right)\cos\left(\frac{\pi }{2}\theta_{2}\right)\left(1+g\left(x\right)\right)$	12	[0,1]	Inverted-Curved
	$f_{2}\left(x\right)=\cos\left(\frac{\pi }{2}\theta_{1}\right)\sin\left(\frac{\pi }{2}\theta_{2}\right)\left(1+g\left(x\right)\right)$			
	$f_{3}\left(x\right)=\sin\left(\frac{\pi }{2}\theta_{1}\right)\left(1+g\left(x\right)\right)$			
	$\theta_{1}=x_{1}.\left(\frac{\pi }{2}\right),\theta_{2}=\frac{\pi }{4.\left(1+g\left(x\right)\right)}.\left(1+2x_{i}.g\left(x\right)\right)$$g\left(x\right)=\sum_{i=3}^{n}{\left(x_{i}\right)}^{0.1}$			
DTLZ7	$f_{1}\left(x\right)=x_{1}$	12	[0,1]	Disconnected
	$f_{2}\left(x\right)=x_{2}$			
	$f_{3}\left(x\right)=\left(1+g\left(x\right)\right).h\left(f_{1},f_{2},g\left(x\right)\right)$			
	$g\left(x\right)=1+\frac{9}{22}\sum_{i=3}^{n}\left(x_{i}\right),h\left(f_{1},f_{2},g\left(x\right)\right)=3-\sum_{i=1}^{2}\left(\frac{f_{i}}{1+g}\left(1+\text{si}n\left(3\pi f_{i}\right)\right)\right)$
(iii) Real-World Engineering Design Problems [94,95]: This category comprises five practical engineering challenges, namely the 4-bar truss design (RW1), disk brake design (RW2), speed reducer design (RW3), welded beam design (RW4), and cantilever beam design (RW5). These problems provide a concrete framework for appraising MORCOA's practical utility and performance in real-world engineering contexts.

The design of honeycomb heat sinks (RW6) is particularly highlighted as a distinct subsection within this study [96]. This specific problem encapsulates the intricacies and challenges associated with optimizing thermal management solutions in engineering. Through the honeycomb heat sink design problem, the study showcases MORCOA's adeptness in navigating complex design parameters to achieve optimal thermal performance, underlining the algorithm's relevance and adaptability to engineering optimization tasks.

### 4.1 Results – ZDT and DTLZ functions

This research undertook a comprehensive evaluation of the effectiveness of various optimization algorithms, utilizing a suite of performance indicators to ensure a thorough analysis. These indicators include HV, GD, IGD, SD, SP, and RT. For a more intuitive hold of these metrics, Fig. 2 provides detailed explanations and visual representations of each. In the proposed study, all algorithms are executed individually 30 times, enabling a fair statistical evaluation. Wilcoxon signed-rank test (WSRT) was conducted at a significance level of 0.05 on which the respective algorithms have a better, a worse and an equal performance of MOIPSO, NSGA-II, MOGBO, and MOAGDE with respect to the proposed MORCOA. The outcomes were denoted using the symbols “+/−/ = ,” representing superior, inferior, and equivalent performance, respectively. This statistical method was applied to contrast the performance of various optimizers across a range of test scenarios, aiming to identify conditions under which these optimizers demonstrated superior, inferior, or comparable effectiveness in solving MOO problems. The comparative study encompassed the proposed MORCOA and several other notable multi-objective optimization algorithms, including (i) Multi-Objective Adaptive Guided Differential Evaluation (MOAGDE): An advanced algorithm that adapts and guides the search process to navigate the solution space efficiently. The MOAGDE locates Pareto optimal solutions for MOOPs with different decision/objective spaces. The MOAGDE was advanced by reshaping the adaptive guided DE algorithm for MOO; (ii) Improved Multi-Objective Particle Swarm Optimization (MOIPSO): A variant of the PSO framework, enhanced for better performance in multi-objective contexts. In each cycle of its operation, the algorithm conducts an assessment of the external population's variety. Based on the outcome, it decides on executing mutation processes on the external population. Furthermore, it selects varying strategies for updating the particle population, guided by the assessed values. This approach is seamlessly integrated with the principles of CD and non-dominant sorting, ensuring a synergistic interaction that enhances the effectiveness of the algorithm; (iii) Multi-Objective Gradient-Based Optimizer (MOGBO): Utilizes Newton's search rule to improve the search process, aiming for a more directed approach to finding optimal solutions; (iv) NSGA-II (Non-dominated Sorting Genetic Algorithm II): NSGA-II stands out in the domain of MOO for its rapid and superior strategy. It utilizes a non-dominated sorting method to rank solutions according to their dominance hierarchy. Esteemed for its capability to rapidly approach the Pareto front, NSGA-II simultaneously ensures the preservation of solution diversity, marking its efficiency and effectiveness in optimization tasks. This research meticulously compares the performance of MORCOA against these algorithms, providing a comprehensive overview and insightful analysis of each algorithm's capabilities and performance metrics in tackling multi-objective optimization challenges. The control parameters of all selected algorithms are provided in the *Appendices*. All the algorithms are executed 30 times individually for a fair comparison. The performance of the proposed MORCOA is obvious in both the mean (MEAN) and standard deviations (STD) values listed in all metric tables.

The summarized performance of various algorithms on ZDT and DTLZ test functions reveals MORCOA as a standout performer. In the context of GD metrics, as listed in Table 3, MORCOA demonstrates superior results across the board, showcasing the smallest values in the majority of the test cases, indicative of its effectiveness in minimizing the generational distance. This is especially evident in the ZDT1 and ZDT2 problems, where MORCOA achieves significantly lower GD values compared to other algorithms like NSGA-II, MOIPSO, MOAGDE, and MOGBO. Its performance suggests an exceptional capability in converging towards the Pareto front while ensuring a diverse solution set.

**Table 3:** GD metrics of ZDT and DTLZ test functions.

Problem	M	NSGA-II	MOIPSO	MOAGDE	MOGBO	MORCOA
ZDT1	2	7.2045e-4 (2.04e-4) -	5.5523e-5 (5.55e-5) -	2.3922e-4 (3.96e-5) -	1.6882e-4 (8.79e-5) -	7.0812e-6 (2.67e-7)
ZDT2	2	4.9576e-4 (2.39e-4) -	5.9375e-6 (5.04e-7) =	8.3658e-4 (2.65e-4) -	2.2611e-4 (9.22e-5) -	6.4197e-6 (6.96e-7)
ZDT3	2	1.6936e-4 (5.03e-5) -	5.1251e-5 (2.29e-5) =	9.2672e-4 (2.81e-4) -	8.6095e-5 (3.46e-5) =	7.6050e-5 (1.86e-5)
ZDT4	2	1.1637e-4 (7.46e-5) =	6.4948e-1 (4.03e-1) -	1.4447e-3 (8.41e-4) -	2.0847e-1 (1.76e-1) -	7.3824e-5 (5.75e-5)
ZDT6	2	1.3663e-5 (1.25e-5) -	1.1629e-1 (6.98e-2) -	7.2840e-4 (2.84e-4) -	9.0456e-3 (1.49e-2) =	4.9198e-6 (3.72e-7)
DTLZ1	3	9.8846e-4 (8.37e-4) =	8.5042e+0 (5.21e-1) -	3.2292e-4 (8.31e-5) =	5.5921e+0 (3.65e+0) -	2.8705e-4 (2.20e-5)
DTLZ2	3	1.7807e-3 (5.13e-4) -	3.8883e-3 (9.00e-4) -	6.9905e-4 (1.20e-6) =	7.3342e-3 (2.65e-3) -	6.9731e-4 (3.69e-6)
DTLZ3	3	8.2689e-3 (1.40e-2) =	2.5499e+1 (6.60e-1) -	1.1011e-3 (3.03e-4) =	1.9429e+1 (6.52e+0) -	1.2231e-3 (5.56e-4)
DTLZ4	3	1.9255e-3 (6.01e-4) -	2.4031e-3 (8.36e-4) -	7.0052e-4 (5.32e-6) =	7.3359e-4 (8.51e-4) =	6.3345e-4 (8.85e-5)
DTLZ5	3	6.0146e-4 (2.34e-4) =	5.6797e-4 (5.40e-4) =	9.9958e-6 (4.54e-7) +	4.1075e-4 (8.45e-5) =	3.9939e-4 (6.29e-5)
DTLZ6	3	6.2645e-6 (5.76e-7) =	1.6142e-4 (7.46e-5) -	1.2584e-2 (2.52e-2) -	7.1633e-6 (9.74e-7) =	6.6348e-6 (8.21e-7)
DTLZ7	3	5.3928e-3 (1.83e-3) =	8.2969e-3 (2.06e-3) =	1.6427e-2 (4.03e-3) -	1.0714e-2 (3.40e-3) =	5.7705e-3 (3.11e-3)
+/−/ =		0/6/6	0/8/4	1/7/4	0/6/6	

The insights from the IGD metrics, as listed in Table 4, across ZDT and DTLZ test functions highlight MORCOA's commendable performance. The proposed MORCOA demonstrates a mix of superior, equivalent, and, in fewer cases, inferior performances compared to other algorithms like NSGA-II, MOIPSO, MOAGDE, and MOGBO. Specifically, MORCOA shows outstanding results in ZDT2 and ZDT6 problems, where it outperforms its counterparts with the lowest IGD values, signalling its strength in maintaining a good balance between convergence and diversity towards the Pareto front.

**Table 4:** IGD metrics of ZDT and DTLZ test functions.

Problem	M	NSGA-II	MOIPSO	MOAGDE	MOGBO	MORCOA
ZDT1	2	1.2703e-2 (3.20e-4) -	8.2757e-3 (2.04e-4) +	7.8680e-3 (1.48e-4) +	1.0942e-2 (1.97e-3) -	9.8560e-3 (9.95e-7)
ZDT2	2	1.0230e-2 (5.90e-4) -	8.4165e-3 (8.80e-5) -	8.9290e-3 (6.75e-4) -	9.8612e-3 (2.19e-4) -	7.6915e-3 (3.62e-8)
ZDT3	2	1.0944e-2 (1.27e-4) +	9.9319e-3 (8.30e-5) +	2.8845e-2 (1.27e-2) -	1.1069e-2 (8.60e-4) =	1.2028e-2 (6.04e-4)
ZDT4	2	9.8920e-3 (3.25e-4) -	4.1352e+0 (2.60e+0) -	1.3027e-2 (3.83e-3) -	1.1744e+0 (9.79e-1) -	7.8954e-3 (6.67e-5)
ZDT6	2	7.7923e-3 (6.06e-4) -	7.9355e-3 (4.54e-4) -	7.7894e-3 (1.02e-3) -	7.9693e-3 (4.64e-4) -	6.0637e-3 (4.84e-8)
DTLZ1	3	4.2065e-2 (3.63e-3) -	3.2575e+1 (7.68e+0) -	3.1019e-2 (1.71e-4) =	2.8930e+0 (3.16e+0) -	3.0959e-2 (8.47e-5)
DTLZ2	3	9.5462e-2 (1.01e-3) -	9.0846e-2 (2.33e-3) -	8.1353e-2 (6.82e-7) =	1.0439e-1 (9.60e-3) -	8.1353e-2 (1.77e-6)
DTLZ3	3	9.8094e-2 (5.51e-3) -	7.5445e+1 (3.22e+1) -	8.1960e-2 (4.72e-4) =	1.2359e+1 (1.36e+1) -	8.2286e-2 (7.53e-4)
DTLZ4	3	9.5201e-2 (4.64e-3) =	1.4616e-1 (1.60e-2) =	8.1355e-2 (2.61e-6) =	6.0311e-1 (3.96e-1) =	3.1242e-1 (2.67e-1)
DTLZ5	3	1.3081e-2 (1.25e-3) +	1.0258e-2 (5.71e-4) +	5.0781e-2 (4.38e-6) -	1.2411e-2 (6.96e-4) +	2.4186e-2 (3.54e-3)
DTLZ6	3	1.2499e-2 (3.46e-4) +	9.4715e-3 (1.93e-4) +	5.0789e-2 (4.23e-6) -	1.1735e-2 (2.11e-4) +	3.6379e-2 (5.33e-3)
DTLZ7	3	2.4270e-1 (1.39e-1) =	1.3276e-1 (1.70e-2) =	2.2502e-1 (3.12e-2) =	1.5233e-1 (9.92e-3) =	2.6711e-1 (1.44e-1)
+/−/ =		3/7/2	4/6/2	1/6/5	2/7/3	

The HV metric analysis, as listed in Table 5, across ZDT and DTLZ test functions indicates that MORCOA exhibits commendable performance. While it does not universally outperform all other algorithms in every test, its results are consistently competitive, often leading or closely matching the best-performing algorithms. Particularly noteworthy is MORCOA's superior performance in specific problems such as ZDT2, where it achieves the highest HV value, suggesting an effective balance between solution diversity and convergence toward the Pareto front. Similarly, in DTLZ test functions, MORCOA's performance is robust, often matching or closely following the leading scores.

**Table 5:** HV metrics of ZDT and DTLZ test functions.

Problem	M	NSGA-II	MOIPSO	MOAGDE	MOGBO	MORCOA
ZDT1	2	7.1001e-1 (3.32e-4) -	7.1584e-1 (1.69e-4) +	7.1508e-1 (5.35e-4) +	7.1202e-1 (2.90e-3) =	7.1367e-1 (2.99e-6)
ZDT2	2	4.3822e-1 (1.06e-3) -	4.4061e-1 (2.15e-4) -	4.3711e-1 (2.10e-3) -	4.3831e-1 (4.87e-4) -	4.4099e-1 (5.15e-8)
ZDT3	2	5.9737e-1 (1.25e-4) =	5.9804e-1 (3.83e-5) +	6.1872e-1 (4.12e-2) =	5.9761e-1 (3.02e-4) =	5.9735e-1 (2.12e-4)
ZDT4	2	7.1339e-1 (4.71e-4) -	0.0000e+0 (0.00e+0) -	7.0461e-1 (6.23e-3) -	1.4846e-1 (2.42e-1) -	7.1533e-1 (4.69e-4)
ZDT6	2	3.8417e-1 (6.15e-4) -	3.8400e-1 (4.89e-4) -	3.8198e-1 (2.04e-3) -	3.8406e-1 (5.98e-4) -	3.8595e-1 (6.40e-8)
DTLZ1	3	7.9391e-1 (5.84e-3) -	0.0000e+0 (0.00e+0) -	8.2217e-1 (1.49e-3) =	1.0859e-1 (1.46e-1) -	8.2268e-1 (8.20e-4)
DTLZ2	3	5.0034e-1 (1.08e-2) -	5.0843e-1 (6.42e-3) -	5.3811e-1 (3.80e-6) =	4.8492e-1 (9.03e-3) -	5.3812e-1 (4.67e-6)
DTLZ3	3	5.0318e-1 (8.12e-3) -	0.0000e+0 (0.00e+0) -	5.3054e-1 (4.07e-3) =	4.9762e-2 (5.77e-2) -	5.2926e-1 (6.27e-3)
DTLZ4	3	5.0556e-1 (1.16e-2) =	5.1908e-1 (8.00e-3) =	5.3811e-1 (1.16e-5) +	2.8275e-1 (2.22e-1) =	4.3321e-1 (1.21e-1)
DTLZ5	3	1.9524e-1 (3.84e-4) +	1.9673e-1 (4.91e-4) +	1.7312e-1 (3.28e-6) -	1.9491e-1 (4.27e-4) +	1.8576e-1 (2.04e-3)
DTLZ6	3	1.9605e-1 (3.73e-4) +	1.9677e-1 (3.46e-4) +	1.7311e-1 (9.41e-7) -	1.9584e-1 (1.06e-4) +	1.8206e-1 (1.34e-3)
DTLZ7	3	2.4295e-1 (9.36e-3) =	2.5804e-1 (3.74e-3) +	2.1475e-1 (1.33e-2) =	2.4134e-1 (3.27e-3) =	2.3271e-1 (7.92e-3)
+/−/ =		2/7/3	5/6/1	2/5/5	2/6/4	

The analysis of SD metrics, as listed in Table 6, for ZDT and DTLZ test functions highlights MORCOA's commendable performance in maintaining a balanced distribution of solutions across the objective space. While the performance varies across different problems, MORCOA consistently shows either superior or competitive results, particularly shining in scenarios where maintaining a diverse set of solutions is crucial. For instance, in ZDT2 and ZDT4 problems, MORCOA demonstrates significantly better spread metrics compared to other algorithms like NSGA-II, MOIPSO, MOAGDE, and MOGBO, indicating its effectiveness in dispersing solutions evenly along the Pareto front. This is further evidenced in the DTLZ problems, where MORCOA again performs well, especially in DTLZ2 and DTLZ3, showcasing its adaptability and robustness in handling complex multi-objective optimization tasks.

**Table 6:** SD metrics of ZDT and DTLZ test functions.

Problem	M	NSGA-II	MOIPSO	MOAGDE	MOGBO	MORCOA
ZDT1	2	4.3487e-1 (4.19e-2) -	3.1745e-1 (3.93e-2) =	2.5930e-1 (3.36e-3) +	4.9742e-1 (7.58e-2) -	2.9873e-1 (2.38e-4)
ZDT2	2	4.4451e-1 (1.08e-1) -	3.2170e-1 (5.04e-2) -	1.6461e-1 (7.78e-3) -	4.5021e-1 (9.67e-3) -	1.4780e-1 (1.06e-4)
ZDT3	2	4.3451e-1 (6.33e-2) +	3.4276e-1 (5.27e-2) +	5.8171e-1 (9.16e-2) =	4.0807e-1 (2.28e-2) +	5.9475e-1 (2.84e-2)
ZDT4	2	6.0984e-1 (1.04e-1) -	8.1658e-1 (7.36e-2) -	2.5680e-1 (6.71e-3) +	8.1966e-1 (1.54e-1) -	2.9792e-1 (2.77e-3)
ZDT6	2	5.3100e-1 (1.61e-1) -	1.1330e+0 (1.53e-1) -	1.5160e-1 (1.11e-2) -	7.1663e-1 (4.30e-1) -	1.0774e-1 (2.35e-4)
DTLZ1	3	5.7302e-1 (5.80e-2) -	6.1300e-1 (1.04e-1) -	3.0633e-3 (2.56e-3) =	8.0686e-1 (2.25e-1) -	2.2932e-3 (9.43e-4)
DTLZ2	3	5.8124e-1 (5.08e-2) -	4.0291e-1 (3.14e-2) -	1.8442e-1 (4.95e-5) =	3.9089e-1 (1.45e-2) -	1.8447e-1 (8.30e-5)
DTLZ3	3	5.9593e-1 (8.83e-2) -	7.3727e-1 (9.55e-2) -	1.8680e-1 (1.77e-3) =	1.1156e+0 (4.96e-1) -	1.9573e-1 (1.89e-2)
DTLZ4	3	5.2449e-1 (9.27e-2) =	5.9129e-1 (6.43e-2) =	1.8447e-1 (7.56e-5) =	7.1002e-1 (3.35e-1) =	5.9787e-1 (4.78e-1)
DTLZ5	3	5.3730e-1 (4.02e-2) +	3.6205e-1 (3.87e-2) +	2.1515e+0 (8.88e-3) -	3.9040e-1 (6.41e-2) +	9.3888e-1 (2.11e-1)
DTLZ6	3	8.0473e-1 (1.13e-1) +	3.3089e-1 (2.26e-2) +	2.1176e+0 (3.26e-2) -	5.0008e-1 (4.23e-2) +	1.4567e+0 (1.03e-1)
DTLZ7	3	6.1218e-1 (2.78e-2) +	5.3041e-1 (4.13e-2) +	9.9953e-1 (6.16e-3) -	5.1274e-1 (5.21e-2) +	8.1404e-1 (9.50e-2)
+/−/ =		4/7/1	4/6/2	2/5/5	4/7/1	

The analysis of SP metrics, as listed in Table 7, across ZDT and DTLZ test functions, reveals an improved performance profile for MORCOA, highlighting its strengths and areas of competitiveness. In some instances, such as ZDT2 and ZDT6, MORCOA showcases superior performance, achieving the lowest spacing metrics, which indicate a more uniform distribution of solutions across the objective space. This is particularly significant in optimization problems where a well-distributed set of solutions is essential for effective decision-making. However, the performance of MORCOA is not uniformly dominant across all test functions. In scenarios like ZDT1, ZDT3, and DTLZ7, its spacing metrics suggest areas where its performance is on par with or slightly lagging behind other algorithms. This mixed outcome underscores the complexity of multi-objective optimization tasks and the challenge of achieving optimal performance across diverse problem sets. MORCOA's performance in spacing metrics, while varied, demonstrates its ability to maintain a competitive edge in ensuring uniform solution distributions in several key test scenarios.

**Table 7:** SP metrics of ZDT and DTLZ test functions.

Problem	M	NSGA-II	MOIPSO	MOAGDE	MOGBO	MORCOA
ZDT1	2	1.8507e-2 (2.89e-3) =	1.3665e-2 (1.21e-3) +	7.9902e-3 (5.59e-5) +	1.6956e-2 (2.64e-3) +	2.2389e-2 (1.94e-5)
ZDT2	2	1.5718e-2 (2.12e-3) -	1.3397e-2 (9.83e-4) -	9.5609e-3 (4.59e-4) -	1.6123e-2 (1.13e-3) -	8.6954e-3 (2.74e-6)
ZDT3	2	1.8403e-2 (2.36e-3) +	1.5737e-2 (1.90e-3) +	3.2391e-2 (2.03e-3) -	1.7805e-2 (1.11e-3) +	2.3700e-2 (2.27e-3)
ZDT4	2	1.8022e-2 (1.60e-3) =	2.1423e-2 (6.79e-3) =	8.0906e-3 (4.18e-4) +	3.5745e-2 (1.75e-2) -	1.8508e-2 (6.21e-5)
ZDT6	2	1.3490e-2 (2.17e-3) -	1.7261e-1 (7.09e-2) -	7.0428e-3 (8.79e-4) -	6.9157e-2 (9.91e-2) -	4.3206e-3 (7.49e-6)
DTLZ1	3	3.4351e-2 (5.25e-3) -	5.8898e+0 (1.16e+0) -	2.3288e-4 (1.97e-4) =	4.0027e+0 (3.81e+0) -	2.4507e-4 (1.22e-4)
DTLZ2	3	9.1572e-2 (6.27e-3) -	7.8121e-2 (5.62e-3) =	8.3815e-2 (1.19e-5) =	8.3274e-2 (8.68e-3) =	8.3806e-2 (3.93e-5)
DTLZ3	3	8.2129e-2 (2.86e-3) =	1.9457e+1 (3.37e+0) -	8.3179e-2 (1.31e-3) =	3.0445e+1 (8.51e+0) -	8.7534e-2 (6.58e-3)
DTLZ4	3	8.6309e-2 (3.99e-3) =	8.6386e-2 (9.31e-3) =	8.3797e-2 (2.92e-5) =	1.9115e-2 (2.21e-2) =	5.4197e-2 (3.39e-2)
DTLZ5	3	1.9644e-2 (1.04e-3) =	1.7499e-2 (8.36e-4) =	2.7354e-2 (6.86e-4) =	1.5315e-2 (2.16e-3) =	2.5025e-2 (7.60e-3)
DTLZ6	3	2.4221e-2 (1.92e-3) =	1.6515e-2 (8.55e-4) =	9.3349e-2 (1.35e-1) =	2.2796e-2 (1.91e-3) =	2.8287e-2 (1.06e-2)
DTLZ7	3	1.0009e-1 (2.87e-2) =	1.1325e-1 (1.92e-2) =	2.5925e-1 (1.73e-2) -	1.1440e-1 (1.12e-2) =	9.3249e-2 (2.39e-2)
+/−/ =		1/4/7	2/4/6	2/4/6	2/5/5	

The RT metrics, as listed in Table 8, from ZDT and DTLZ test functions indicate that MORCOA showcases a strong performance in terms of computational efficiency. Across the various problems, MORCOA consistently demonstrates lower run times compared to other algorithms like NSGA-II, MOIPSO, MOAGDE, and MOGBO. Especially notable is its performance in the ZDT1, ZDT2, ZDT3, ZDT4, and ZDT6 problems, where MORCOA not only outperforms its competitors in terms of speed but does so with a considerable margin. In the more complex DTLZ test functions, MORCOA's run times remain competitive, often resulting in faster execution compared to NSGA-II and significantly outperforming MOAGDE. The algorithm shows its ability to maintain efficiency even as the problem dimensionality increases, a crucial factor for practical applications of multi-objective optimization. This consistent pattern of superior run-time performance underscores MORCOA's effectiveness as a computationally efficient algorithm capable of delivering quick solutions without sacrificing quality.

**Table 8:** RT metrics of ZDT and DTLZ test functions.

Problem	M	D	NSGA-II	MOIPSO	MOAGDE	MOGBO	MORCOA
ZDT1	2	30	1.1785e+0 (1.05e-1) -	2.7623e+0 (1.63e-1) -	1.7154e+1 (6.32e-2) -	2.0298e+0 (2.64e-1) -	1.0632e+0 (2.81e-2)
ZDT2	2	30	1.2109e+0 (4.22e-2) -	2.7564e+0 (2.63e-2) -	1.7172e+1 (1.26e-1) -	1.7648e+0 (5.03e-2) -	1.0455e+0 (1.73e-2)
ZDT3	2	30	1.1883e+0 (2.41e-2) -	1.6158e+0 (8.43e-2) -	1.7068e+1 (6.01e-2) -	1.7825e+0 (1.22e-2) -	1.1155e+0 (7.90e-2)
ZDT4	2	10	1.0659e+0 (2.14e-2) -	1.1446e+0 (7.62e-2) -	1.6699e+1 (4.16e-2) -	1.5957e+0 (1.51e-2) -	9.6428e-1 (1.16e-2)
ZDT6	2	10	1.0129e+0 (1.03e-2) -	1.1298e+0 (4.19e-2) -	1.6842e+1 (1.49e-1) -	1.5643e+0 (2.83e-2) -	9.9854e-1 (1.25e-2)
DTLZ1	3	7	1.8682e+0 (4.57e-2) -	9.4441e-1 (1.15e-2) +	1.7629e+1 (1.15e-1) -	1.1378e+0 (1.51e-2) +	1.2219e+0 (1.33e-1) +
DTLZ2	3	12	1.7465e+0 (2.26e-2) -	1.7123e+0 (1.09e-1) -	1.7335e+1 (1.35e-1) -	1.1474e+0 (1.27e-2) -	1.0706e+0 (1.80e-2) +
DTLZ3	3	12	1.8467e+0 (6.92e-2) -	8.9961e-1 (3.20e-2) +	1.8134e+1 (5.03e-1) -	1.1186e+0 (3.23e-2) -	1.1110e+0 (2.11e-2) +
DTLZ4	3	12	3.5762e+0 (1.94e+0) -	9.1911e-1 (2.87e-2) +	1.8068e+1 (5.34e-1) -	1.1943e+0 (7.51e-2) -	1.0814e+0 (1.20e-2) +
DTLZ5	3	12	5.2826e+0 (4.15e-1) -	1.5779e+0 (1.13e-1) -	1.8628e+1 (1.24e-1) -	1.2435e+0 (4.62e-2) -	1.1465e+0 (4.96e-2) +
DTLZ6	3	12	5.7018e+0 (2.33e-1) -	2.3764e+0 (8.14e-2) -	1.8633e+1 (3.00e-1) -	1.3318e+0 (7.64e-2) -	1.1775e+0 (6.69e-2) +
DTLZ7	3	22	1.9478e+0 (8.10e-2) -	2.2123e+0 (8.43e-2) -	1.8036e+1 (3.71e-1) -	1.1501e+0 (3.44e-2) +	1.1973e+0 (5.17e-2) +
+/−/ =			0/12/0	3/9/0	0/12/0	0/12/0	

MORCOA emerges as a standout algorithm in the realm of multi-objective optimization based on a thorough evaluation of its performance across a variety of metrics. Its ability is consistently demonstrated through superior results, showcasing its robustness and effectiveness in generating high-quality solutions. Particularly notable is MORCOA's performance in GD and IGD metrics, where it excels in maintaining a balance between convergence towards the Pareto Front (PF) and diversity among solutions. This balance is crucial for effective MOO and is further evidenced in its outstanding results in HV metrics, indicating successful navigation of competing objectives. MORCOA's capabilities extend to SP and SD metrics, where it often outperforms competitors by ensuring a comprehensive exploration of the solution space with evenly distributed solutions. Moreover, its efficiency in the RT metric is increased, demonstrating a swift processing ability that makes it particularly suited for time-sensitive applications. This efficiency, coupled with its adaptability and versatility across various problem types, underscores MORCOA's value in real-world scenarios where quick and reliable solutions are dominant.

**Fig. 6:**
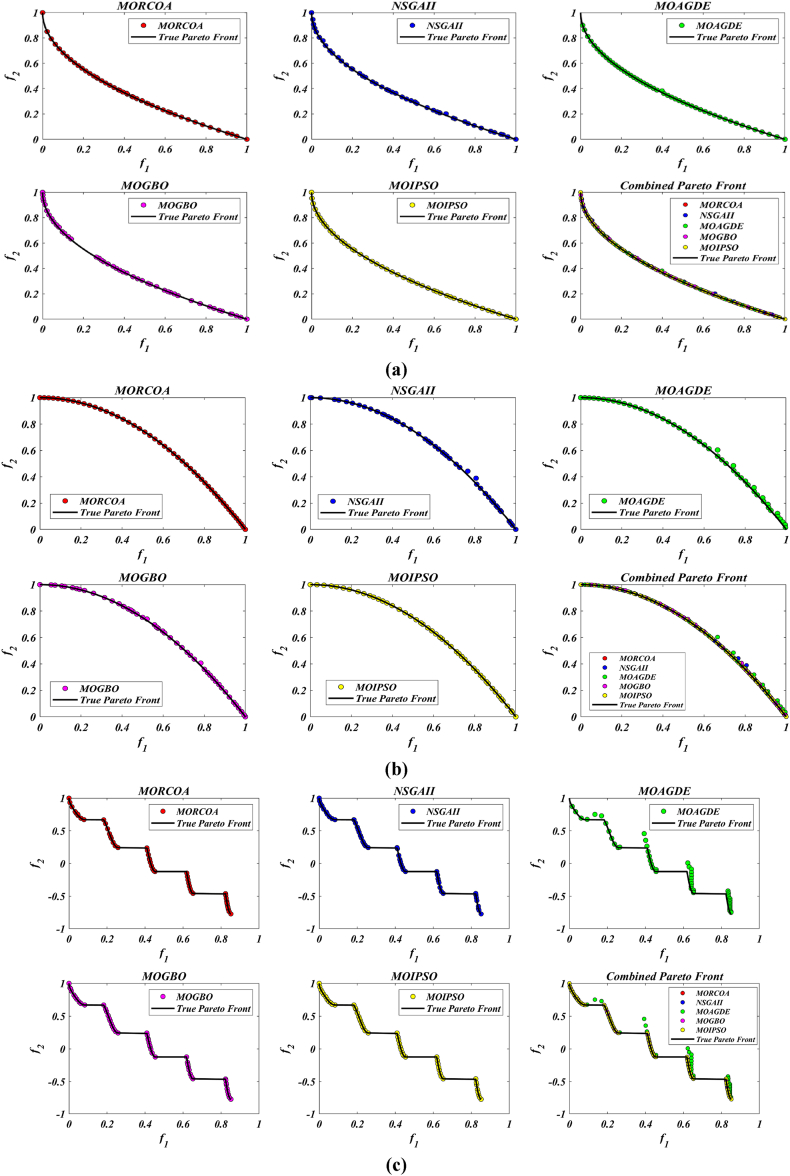

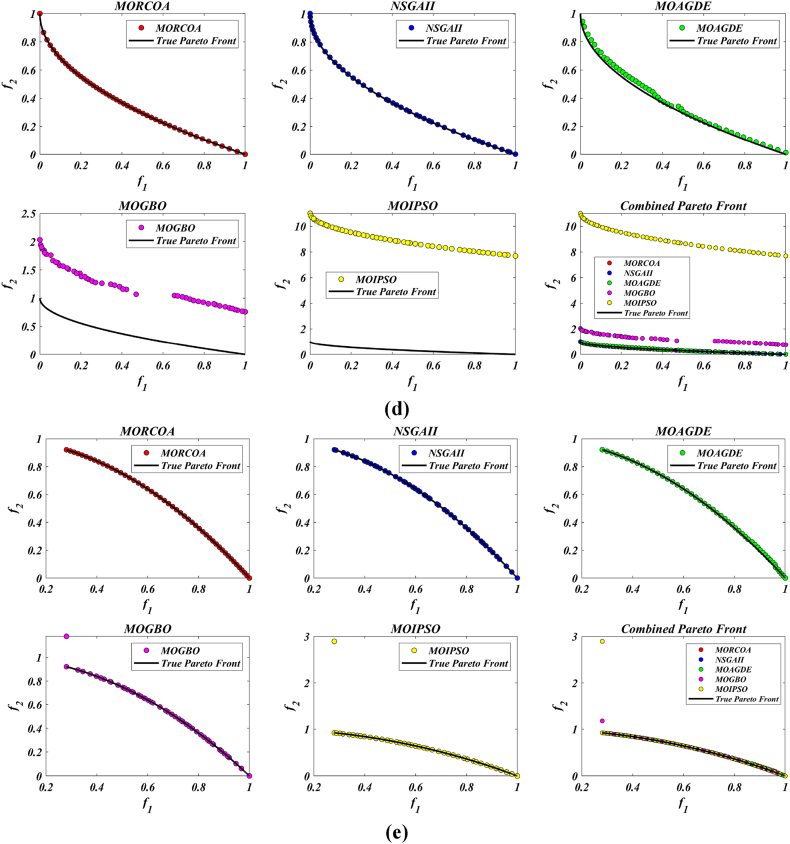
PF obtained by all algorithms for ZDT problems; (a) ZDT1, (b) ZDT2, (c) ZDT3, (d) ZDT4, (e) ZDT6

In this research, the newly introduced MORCOA was examined for its efficacy in constructing Pareto fronts across various ZDT benchmark problems. The comparative analysis positioned MORCOA in a leading stance against a suite of established algorithms, including the NSGA-II, MOIPSO, MOGBO, and MOAGDE. Fig. 6, integral to this study, visually delineates the PFs as generated by each algorithm under consideration. Notably, Fig. 6 comprises individual PF plots for each algorithm alongside a collective PF plot, facilitating a direct comparison of their performance metrics. A significant highlight from the analysis was MORCOA's noticeable accuracy in navigating towards the true PF, a superiority that was markedly observable within the more intricate regions of the objective space. Interestingly, this precise convergence was achieved without the necessity for any parameter tuning within the proposed algorithm, underscoring its inherent robustness and adaptability. Moreover, the PF realized through MORCOA was characterized by a more uniform distribution of solutions. This uniformity is indicative of the algorithm's comprehensive exploration capabilities across the objective space, ensuring that a broad spectrum of viable solutions is considered. In contrast, the comparative algorithms frequently exhibited tendencies towards solution clustering or the presence of significant gaps within their generated PFs. Such discrepancies highlight the limitations of these algorithms in adequately spanning the objective space, thereby highlighting the enhanced performance and efficiency of MORCOA in multi-objective optimization scenarios.

In this study, the MORCOA showcased exceptional adaptability and performance across a range of benchmark problems, notably the ZDT and DTLZ test suites, which are renowned for their complexity and varied PF geometries, including convex, concave, and discontinuous shapes. Unlike its counterparts - MOIPSO, MOGBO, NSGA-II, and MOAGDE - MORCOA demonstrated unparalleled consistency in performance across multiple runs, showcasing less variance in solution quality and indicating higher reliability for multi-objective optimization tasks.

**Fig. 7:**
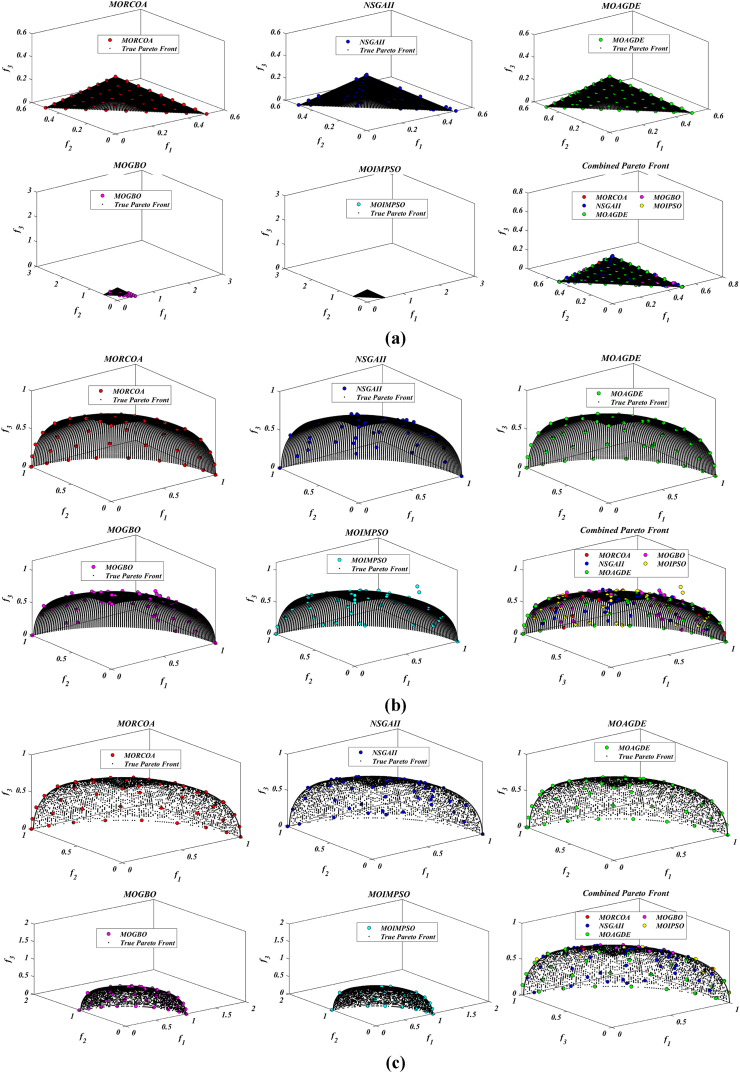

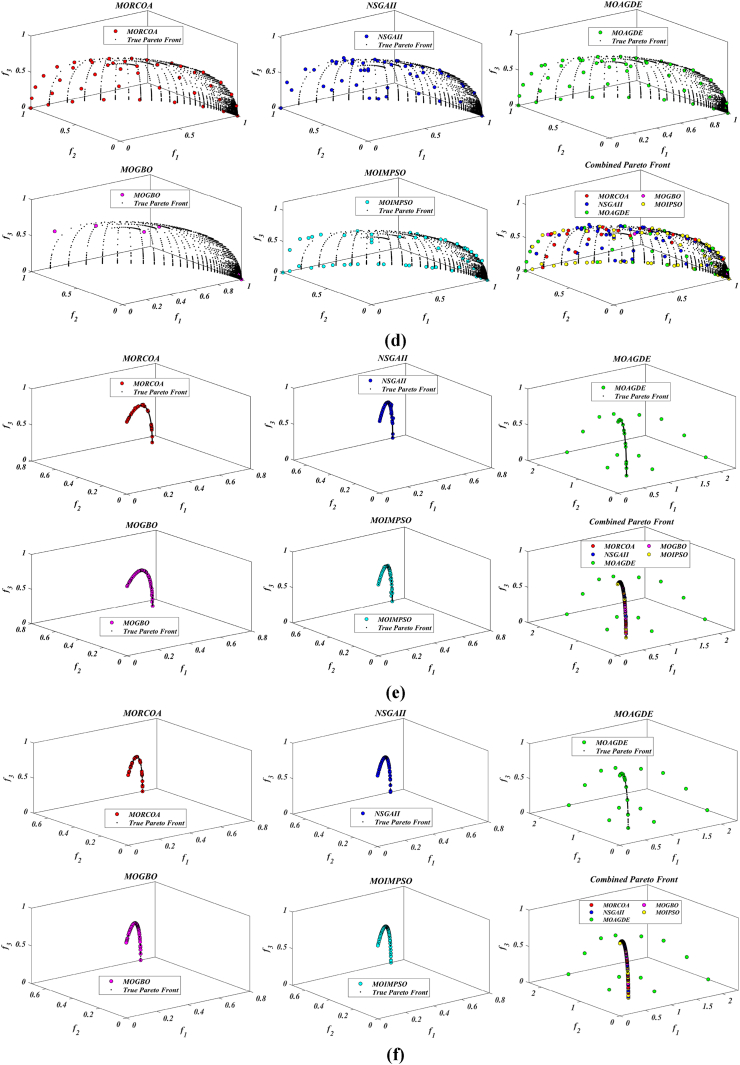

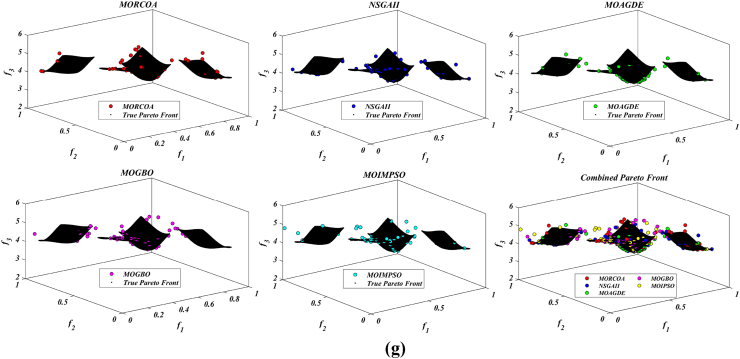
PF obtained by all algorithms for DTLZ problems; (a) DTLZ1, (b) DTLZ2, (c) DTLZ3, (d) DTLZ4, (e) DTLZ5, (f) DTLZ6, (g) DTLZ7

MORCOA's adaptability was particularly highlighted in its handling of the DTLZ problems set with tri-objective challenges, where it not only achieved remarkable precision in converging towards the true PF but also excelled in managing the intricate interdependencies between objective functions. This level of precision in convergence, particularly under the complex conditions presented by tri-objective scenarios, sets MORCOA apart from competing algorithms. The algorithm's sophisticated structure was adept at navigating these complexities, leading to Pareto fronts characterized by superior solution distribution and diversity. Unlike the other algorithms, MORCOA produced PFs that were more uniformly distributed, ensuring a comprehensive exploration of the objective space. This uniformity is vital for capturing the entire spectrum of possible trade-offs between competing objectives. Fig. 7 in the study highlights MORCOA's superiority, showing its ability to generate more accurate and diverse PFs compared to its counterparts. This capability is crucial for understanding the full range of solutions available in MOO scenarios. Moreover, despite its progressive optimization features, MORCOA maintained computational efficiency on par with or exceeding that of the compared algorithms.

This efficiency is particularly important in scenarios with limited computational resources, highlighting MORCOA's practicality in resource-constrained environments. Overall, MORCOA's robustness, adaptability, and computational efficiency underscore its effectiveness and potential as a powerful tool for tackling complex multi-objective optimization problems.

**Fig. 8:**
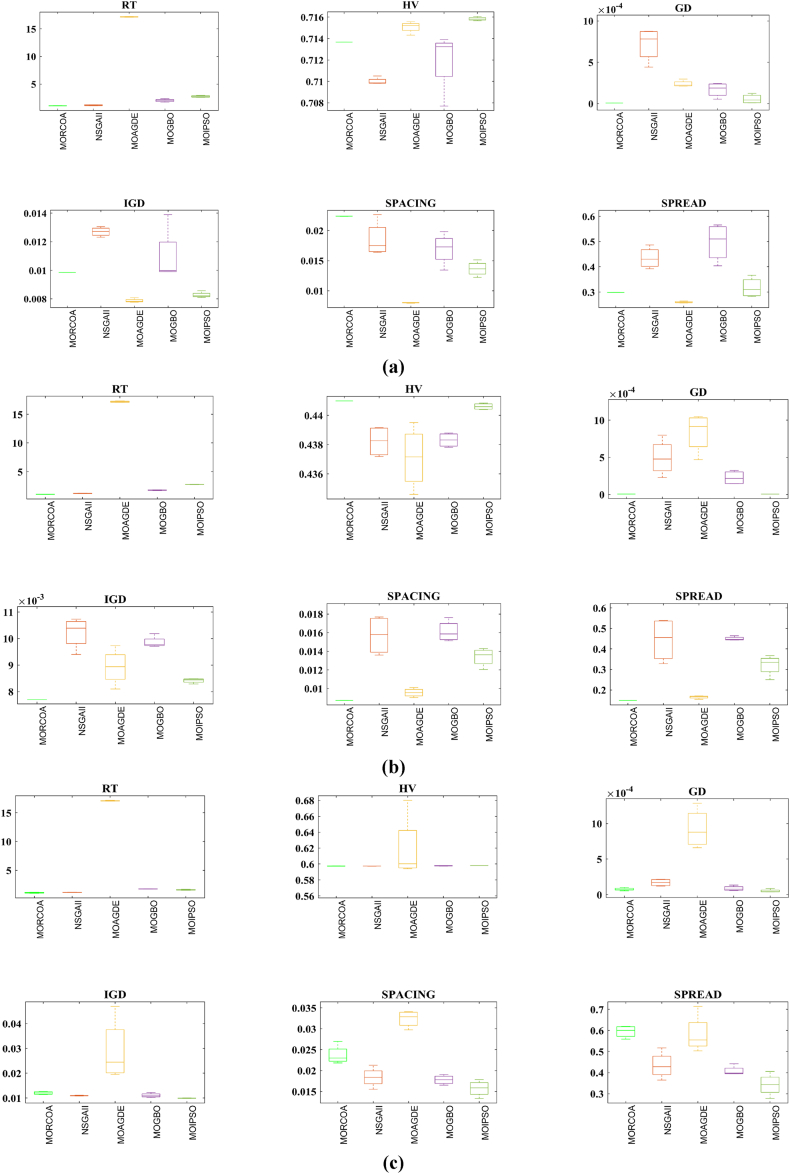

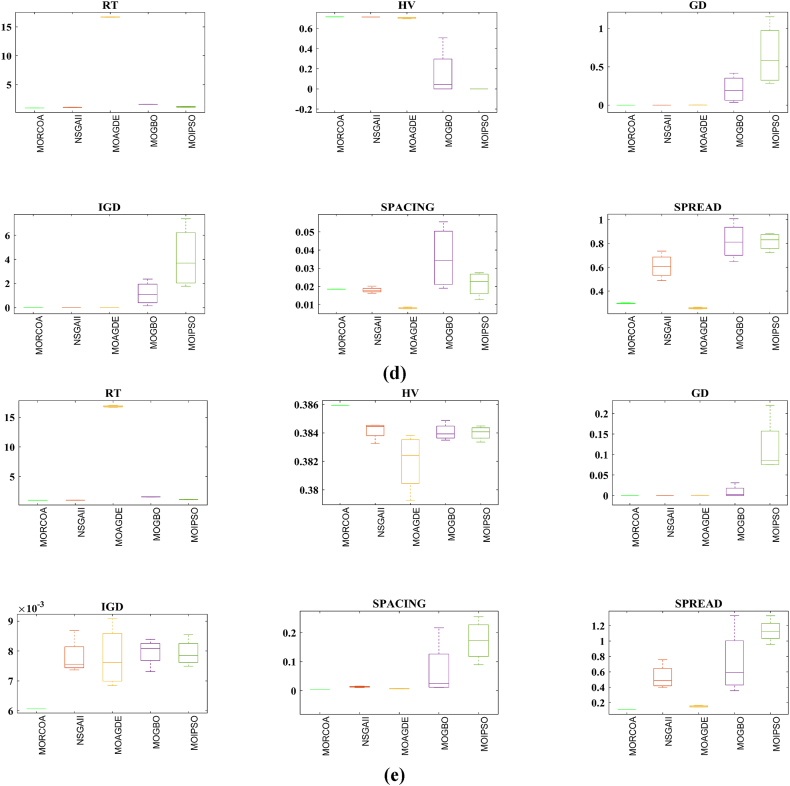
Boxplots obtained for all algorithms for ZDT problems: (a) ZDT1, (b) ZDT2, (c) ZDT3, (d) ZDT4, (e) ZDT6

**Fig. 9:**
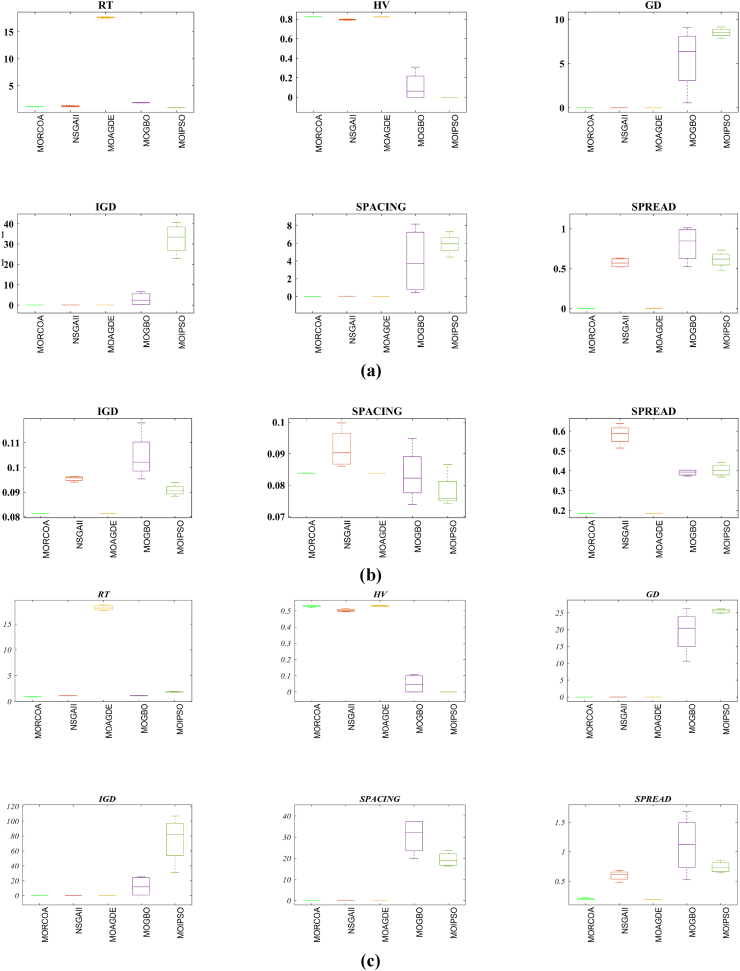

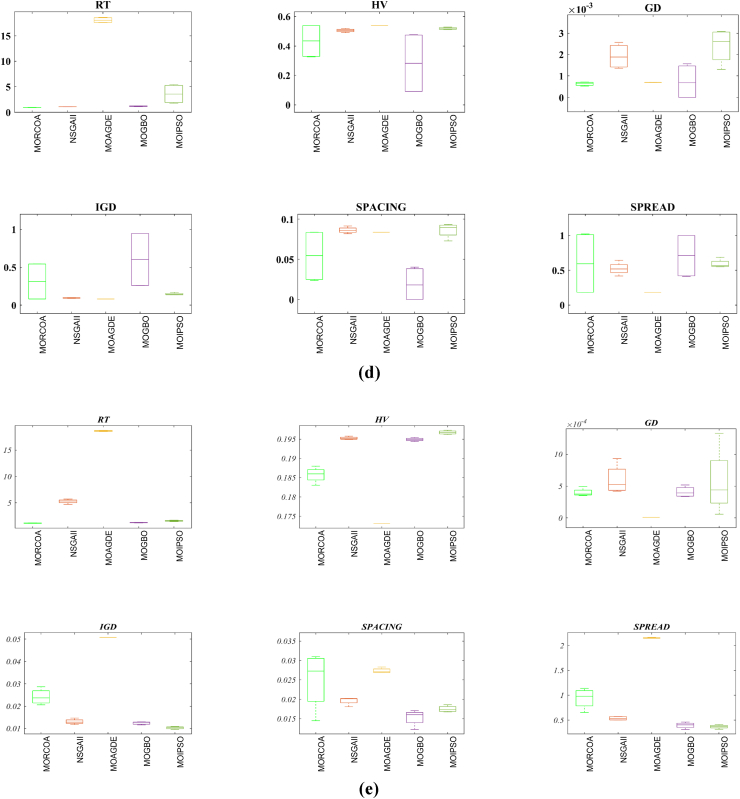

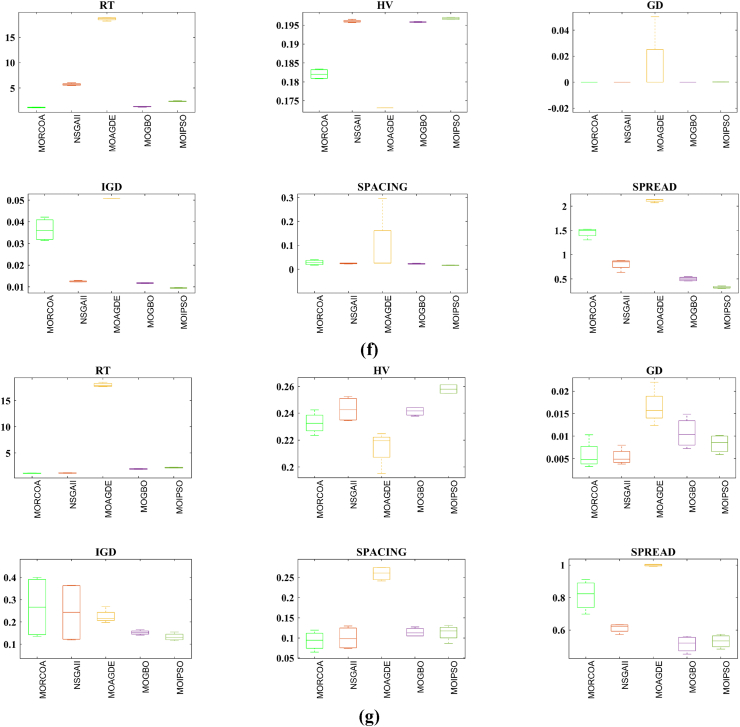
Boxplots obtained for all algorithms for DTLZ problems: (a) DTLZ1, (b) DTLZ 2, (c) DTLZ 3, (d) DTLZ 4, (e) DTLZ 5, (f) DTLZ6, (g) DTLZ7

The visualization of box plots, as shown in Fig. 8, Fig. 9, for various metrics such as RT, SP, SD, HV, GD, and IGD across the ZDT and DTLZ problem sets, respectively, serves as a comprehensive statistical tool to evaluate and compare the performance of the proposed MORCOA against other algorithms. These plots provide a multifaceted view of MORCOA's reliability and efficacy in MOO by illustrating the range, median, and variability of each metric across all tested problems. The RT box plots depict the computational efficiency of MORCOA relative to other algorithms. A lower median and tighter interquartile range (IQR) suggest MORCOA is not only fast but also consistent in its execution time across multiple runs, indicating efficient algorithmic complexity and implementation. SP metrics assess the uniformity in the distribution of solutions along the Pareto front. Box plots showcasing lower medians and smaller IQRs for MORCOA indicate its ability to generate evenly spaced solutions, contributing to its reliability in providing a diverse set of solutions that are well-distributed across the objective space. The SD box plots evaluate the extent to which the solutions cover the true Pareto front. MORCOA's performance in this metric, indicated by lower variability and a median close to optimal values, demonstrates its effectiveness in exploring the objective space thoroughly and capturing a wide range of optimal solutions. HV represents the size of the space covered by the Pareto front relative to a reference point.

Box plots with higher medians and narrow IQR for MORCOA signify its superior ability to achieve solutions that not only converge to the true Pareto front but also cover a substantial portion of the objective space, highlighting its robustness and reliability in capturing the true trade-offs between objectives. GD and IGD box plots provide insights into the convergence and diversity of the solutions, respectively. For MORCOA, lower GD and IGD values indicate a strong convergence towards the true Pareto front while maintaining a diverse set of solutions. This balance between convergence and diversity underscores MORCOA's reliability in accurately and comprehensively addressing MOO problems. The box plot visualization collectively argues for MORCOA's reliability in multi-objective optimization. The algorithm demonstrates not only efficiency and speed (as seen in RT plots) but also excels in providing high-quality solutions that are both diverse and well-distributed (SP, SD), cover a significant portion of the objective space (HV), and closely approximate the true Pareto front with a balanced set of solutions (GD, IGD). The consistency in MORCOA's performance across a wide range of problems, as indicated by the limited variability in the box plots, further cements its reliability and effectiveness as a robust tool for solving complex MOO problems.

### 4.2 Real-world engineering problems

In this comprehensive evaluation, the capabilities of the proposed MORCOA are rigorously examined across a range of difficult engineering design challenges. These real-world scenarios, representative of the multimodal and multi-objective problems frequently encountered within the engineering domain, serve as a testament to the complexity and practical significance of the tasks at hand. The specific engineering problems scrutinized in this study include (i) 4-bar truss design problem, (ii) welded beam design problem, (iii) disk brake design problem, (iv) cantilever beam design problem, (v) speed reducer design problem, and (vi) honeycomb heatsink design problem. Each of these design problems showcases a distinct aspect of engineering optimization, pushing the boundaries of MORCOA to balance multiple objectives within a constrained solution space effectively. The algorithm's performance across these diverse scenarios reveals its utility and effectiveness as a powerful tool for attempting the multi-dimensional optimization tasks inherent in engineering applications. Through this rigorous assessment, MORCOA's proficiency in navigating the complex landscape of engineering design problems is intensely demonstrated, reinforcing its value as an adaptable and reliable benefit for multi-objective optimization problems.

#### 4.2.1 Four-bar truss design problem

In the analysis of the 4-bar truss optimization scenario delineated in source [73], the objective functions $f_{1}$ and $f_{2}$ target the minimization of structural volume and nodal displacement, respectively. This optimization conundrum is critical within the domain of structural engineering, emphasizing the requisites for material utilization efficiency and structural stability. The problem is defined by four design variables $x_{1}$ to $x_{4}$, corresponding to the cross-sectional areas of truss members 1 through 4, which are integral in modulating both the aggregate structural volume and displacement under operational loads. The minimization of the structural volume, denoted by $f_{1}$, entails reducing the material footprint by optimizing the variables related to the cross-sectional areas and the linear dimensions of the truss members, thus directly affecting the volume, as articulated in Eq. (28). Concurrently, the reduction of nodal displacement, encapsulated in $f_{2}$, is pivotal for ensuring structural efficacy and resilience, also formalized in Eq. (28). This dual-faceted optimization strategy underscores the intricate interplay between achieving material efficiency and ensuring structural integrity within engineering design parameters.

(28) $$\begin{array}{c} \text{Minimize}:f_{1}\left(x\right)=200\left(2x_{1}+\sqrt{2x_{2}}+\sqrt{x_{3}}+x_{4}\right)\\ f_{2}\left(x\right)=0.01\left(\frac{2}{x_{1}}+\frac{2\sqrt{2}}{x_{2}}\right) \end{array}\}$$

The displacement experienced by the truss arises from its structural reaction to applied loads, with the cross-sectional areas of its members playing a significant role in determining this response. This displacement can be quantified through the application of structural analysis equations that take into account the truss's geometry, the properties of the materials used, and the specific loading conditions it faces. The design variables $x_{1}$, $x_{2}$, $x_{3}$, and $x_{4}$ represent the cross-sectional areas of the truss's four members, respectively. The values assigned to these variables are critical, as they not only dictate the overall structural volume of the truss but also have a direct impact on its displacement under load. The process of designing the truss is governed by a set of constraints, as outlined in Eq. (29).

(29) $$1.4142\leq x_{2},x_{3}\leq 3;\;1\leq x_{1},x_{4}\leq 3$$

**Fig. 10:**
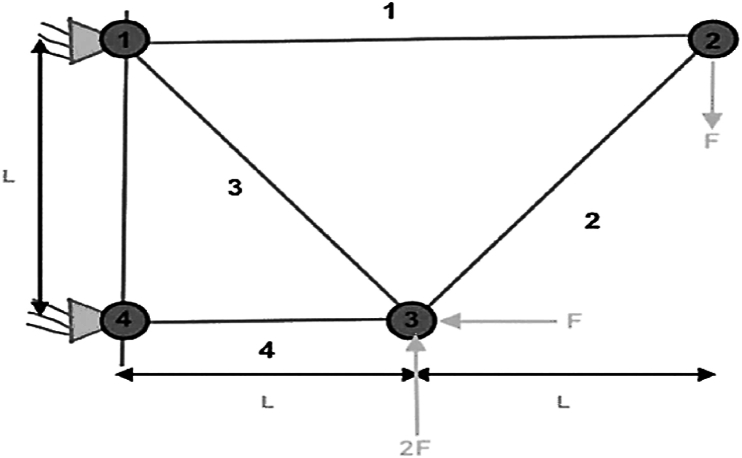
4-bar truss design.

These constraints typically encompass limitations on the stress experienced by each member, a cap on the allowable displacement of the truss as a whole, and additional considerations for the prevention of buckling in members under compression. Such constraints ensure that the final truss design maintains both structural integrity and functionality under the prescribed loading conditions. Fig. 10 presents a diagrammatic view of the truss, detailing the layout of its components and delineating how the design variables $x_{1}$, $x_{2}$, $x_{3}$, and $x_{4}$ relate to each member.

#### 4.2.2 Disk brake design problem

**Fig. 11:**
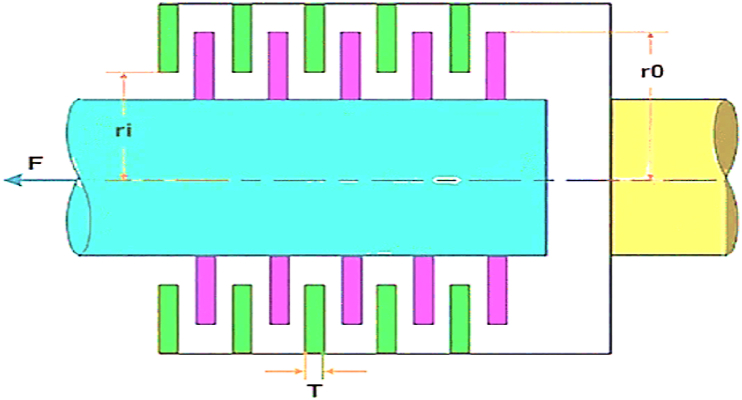
Disk brake design.

The disk brake design challenge, characterized by mixed constraints, was delineated by Osyczka and Kundu [97] within the domain of automotive engineering, aiming at the dual optimization of disk brake performance and efficiency, as depicted in Fig. 11. This issue centralizes on reducing the stopping time $f_{1}$ and the mass $f_{2}$ of the disk brake, essential for augmenting both the safety and operational efficacy of braking systems. The disk's inner radius $x_{1}$ plays a pivotal role in determining thermal capacity and structural resilience, while its outer radius $x_{2}$ is key to modulating braking torque and heat dissipation rates. Additionally, the engaging force $x_{3}$ is intrinsically linked to the exerted braking force, thereby influencing the stopping time. The number of friction surfaces $x_{4}$ directly impacts the brake's frictional interface, affecting both stopping time and thermal efficiency.

Reducing the stopping time is crucial for vehicular safety; facilitating an efficient halt of the vehicle over minimal distances while decreasing the brake mass contributes to the overall reduction in vehicle weight. This reduction is beneficial for enhancing fuel economy and vehicle performance. The defined objective functions and associated constraints are outlined as follows.

(30) $$\begin{array}{c} \text{Minimize}:f_{1}\left(x\right)\;=4.9{\left(10\right)}^{\left(-5\right)}\left(x_{2}^{2}-x_{1}^{2}\right)\left(x_{4}-1\right)\\ f_{2}\left(x\right)=\left(9.82{\left(10\right)}^{\left(6\right)}\right))\left(x_{2}^{2}-x_{1}^{2}\right)/\left(x_{2}^{3}-x_{1}^{3}\right)x_{4}x_{3}) \end{array}\}$$

Subjectedto:

(31) $$\begin{array}{c} g_{1}\left(x\right)\;=20+x_{1}-x_{2}\\ g_{2}\left(x\right)\;=2.5+\left(x_{4}+1\right)-30\\ g_{3}\left(x\right)=x_{3}/\left(3.14{\left(x_{2}^{2}-x_{1}^{2}\right)}^{2}\right)-0.4\\ g_{4}\left(x\right)=\left(2.22{\left(10\right)}^{-3}x_{3}\left(x_{2}^{3}-x_{1}^{3}\right)\right)/{\left(x_{2}^{2}-x_{1}^{2}\right)}^{2}-1\\ g_{5}\left(x\right)\;=900-2.66\left(0.01x_{3}x_{4}\left(x_{2}^{3}-x_{1}^{3}\right){/\left(x_{2}^{2}-x_{1}^{2}\right)}^{2}\right) \end{array}\}$$

The limits are provided in Eq. (32).

(32) $$\begin{array}{c} 55\leq x_{1}\leq 80,75\leq x_{2}\leq 110\\ 1000\leq x_{3}\leq 3000,2\leq x_{4}\leq 20 \end{array}\}$$

The Pareto optimal front for this problem is convex and continuous, showing a direct trade-off between the stopping time and the brake mass. This means that as one objective gets better, the other worsens consistently and measurably. This issue serves as a typical example of MOO in engineering, highlighting the need to find a balance between performance (stopping time) and practical aspects (mass), considering the limits set by physical and mechanical realities.

#### 4.2.3 Speed reducer design problem

**Fig. 12:**
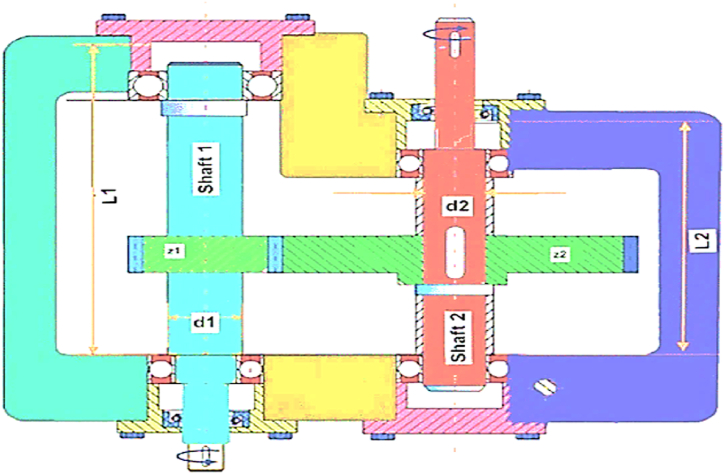
Speed reducer design.

The speed reducer design problem, detailed in Ref. [65] and depicted in Fig. 12, represents a quintessential multi-objective optimization issue within mechanical engineering, aiming to reduce both the weight $f_{1}$ and stress $f_{2}$ of a speed reducer. These objectives are vital for improving the gear system's efficiency and longevity. The design involves seven decision variables that significantly influence the system's performance and structural resilience. Variables such as the gear face width $x_{1}$ and teeth module $x_{2}$ dictate the gear's physical dimensions and operational behaviour, including load capacity and gear teeth spacing. The number of teeth on the pinion $x_{3}$, an integer variable, defines the gear ratio and the pinion's mechanical leverage. The distances between bearings $x_{4}$ and $x_{5}$ determine load distribution and the assembly's mechanical stability, while the diameters of shafts 1 and 2 ($x_{6}$ and $x_{7}$) are critical for ensuring structural integrity and torque transmission capabilities of the respective shafts. This problem exemplifies the balance needed between performance enhancement and practical design constraints in engineering optimizations.

The objective of this problem is to reduce the speed reducer's weight to increase efficiency and lower material costs while also minimizing stress to ensure the gear system's durability and reliability under load. The objective functions and constraints are outlined as follows.

(33) $$\begin{array}{c} \text{Minimize}:f_{1}\left(x\right)=0.7854x_{1}x_{2}^{2}\left(3.3333\left(x_{3}^{2}+14.9334x_{3}\right)-43.0934\right)-1.508x_{1}\left(x_{6}^{2}+x_{7}^{2}\right)\\ f_{2}\left(x\right)=\left({\sqrt{\left(745x_{4}/x_{2}x_{3}\right)}}^{2}+19.9e6\right)/\left(0.1x_{6}^{3}\right) \end{array}\}$$

Subjectedto:

(34) $$\begin{array}{c} g_{1}\left(x\right)=27/\left(x_{1}x_{2}^{2}x_{3}\right)-1\\ g_{2}\left(x\right)=397.5/\left(x_{1}x_{2}^{2}x_{3}^{2}\right)-1\\ g_{3}\left(x\right)=(1.93x_{4}^{3}/\left(x_{2}x_{3}x_{6}^{4}\right)-1\\ g_{4}\left(x\right)=(1.93x_{5}^{3}/\left(x_{2}x_{3}x_{7}^{4}\right)-1\\ g_{5}\left(x\right)=\left({\sqrt{\left(745x_{4}/x_{2}x_{3}\right)}}^{2}+16.9e6\right)/\left(110x_{6}^{3}\right)-1\\ g_{6}\left(x\right)=\left({\sqrt{\left(745x_{4}/x_{2}x_{3}\right)}}^{2}+157.5e6\right)/\left(85x_{7}^{3}\right)-1\\ g_{7}\left(x\right)=\left(x_{2}x_{3}/40\right)1 \end{array}\}$$

The related parameters of this design are given as follows. $\sigma =\frac{504000}{x_{4}x_{3}^{2}}$, $\tau =\sqrt{\alpha^{2}+2\alpha \beta \left(\frac{x_{2}}{2D}\right)+\beta^{2}}$, $P=tmpf\sqrt{\left(x_{3}^{2}\left(\frac{x_{4}^{6}}{36}\right)\right)\left(1-x_{3}\left(\frac{\sqrt{\left(\frac{30}{48}\right)}}{28}\right)\right)}$, and $tmpf=4.013\left(\frac{30\left(10^{6}\right)}{196}\right)$.

#### 4.2.4 Welded beam design problem

**Fig. 13:**
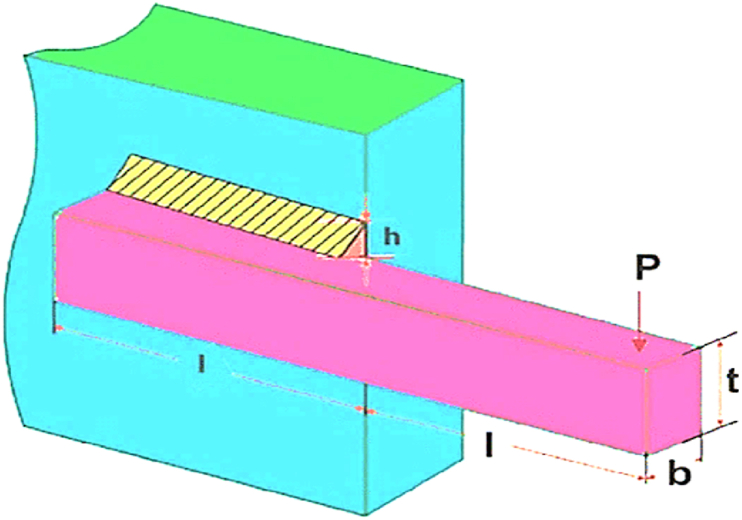
Welded beam design.

The welded beam design problem, introduced by Deb [82], stands as a significant case in structural optimization, focusing on minimizing fabrication cost $f_{1}$ and beam deflection $f_{2}$. These objectives are vital for ensuring cost-effectiveness and maintaining structural integrity. Illustrated in Fig. 13, the design involves four decision variables: the weld thickness $x_{1}$, affecting joint strength and cost; the clamped bar length $x_{2}$, influencing beam dimensions and stability; the bar height $x_{3}$, crucial for load-bearing and deflection, and the bar thickness $x_{4}$, impacting structural behaviour. The challenge aims to reduce material and labour costs while ensuring the beam's structural efficiency and durability under operational loads with specified objective functions and constraints, as follows.

(35) $$\begin{array}{c} \text{Minimize}:f_{1}\left(x\right)=1.10471*x_{1}^{2}*x_{2}+0.04811*x_{3}*x_{4}*\left(14.0+x_{2}\right)\\ f_{2}\left(x\right)=65856000/\left(30\times 10^{6}*x_{4}*x_{3}^{3}\right) \end{array}\}$$

Subjectedto:

(36) $$\begin{array}{c} g_{1}\left(x\right)=\tau -13600\\ g_{2}\left(x\right)=\sigma -30000\\ g_{3}\left(x\right)=x_{1}-x_{4}\\ g_{4}\left(x\right)=6000-P \end{array}\}$$

The limits are provided in Eq. (37).

(37) $$0.125\leq x_{1}\leq 5,0.1\leq x_{2}\leq 10,0.1\leq x_{3}\leq 10,0.125\leq x_{4}\leq 5$$

The related parameters are given as follows. $D=sqrt\left(\frac{x_{2}^{2}}{4}+\frac{{\left(x_{1}+x_{3}\right)}^{2}}{4}\right)$, $\sigma =\sqrt{\left(\alpha^{2}+2*\alpha *\beta *\frac{x_{2}}{2*D}+\beta^{2}\right)}$, $Q=6000*\left(14+\frac{x_{2}}{2}\right)$, $\alpha =\frac{6000}{\sqrt{2}*x_{1}*x_{2}}$, $P=tmpf*sqrt\left(x_{3}^{2}*\frac{x_{4}^{6}}{36}\right)*\left(1-x_{3}*\frac{\sqrt{30/48}}{28}\right)$, $J=2*\left(x_{1}*x_{2}*\sqrt{2}*\left(\frac{x_{2}^{2}}{12}+\frac{{\left(x_{1}+x_{3}\right)}^{2}}{4}\right)\right)$, $\beta =Q*\frac{D}{J}$, $\sigma =\frac{504000}{x_{4}*x_{3}^{2}}$, and $tmpf=4.013*\frac{30*10^{6}}{196}$.

#### 4.2.5 Cantilever beam design

**Fig. 14:**
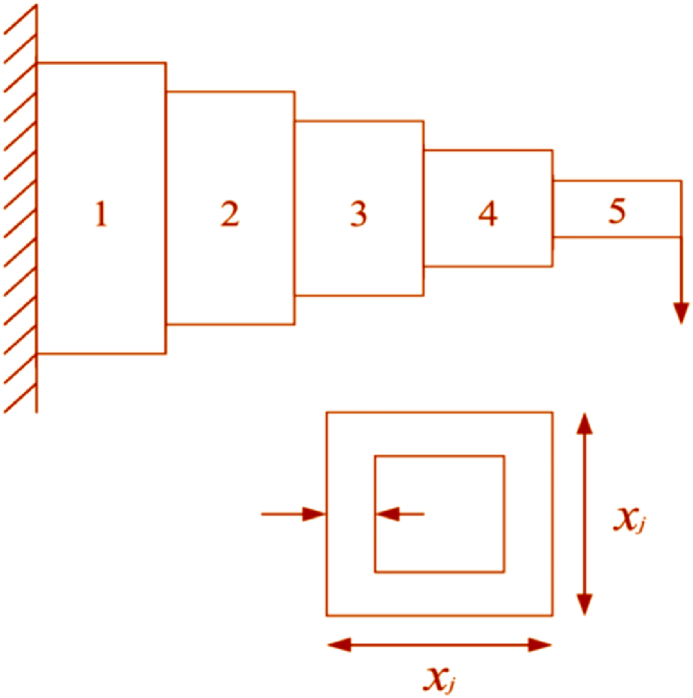
Cantilever beam design.

This study addresses the cantilever beam design problem, a core issue in structural engineering, characterized by three design variables and two objectives under two specific loading scenarios, depicted in Fig. 14 [65]. The variables include the beam's width $x_{1}$, height $x_{2}$, and total length $x_{3}$, all measured in inches. The goals are to minimize the beam's weight for material efficiency and application versatility and to reduce the strain energy, which influences the beam's deflection and structural integrity. The loading conditions considered are: (i) an end load causing maximum bending stress σ, and (ii) a torque load inducing shear stress τ at the beam's root. The optimization challenge integrates these dimensions and objectives within the framework of the specified loading conditions, aiming to enhance the beam's performance and durability. The fitness functions and constraints are as follows.

(38) $$\begin{array}{c} \text{Minimize}:f_{1}\left(x\right)=0.25\times \rho \times \pi \times {x_{1}}^{2}x_{2}\\ f_{2}\left(x\right)=\frac{64\times P\times {x_{2}}^{3}}{3\times E\times \pi \times {x_{1}}^{4}} \end{array}\}$$

Subjectedto:

(39) $$\begin{array}{c} g_{1}\left(x\right)=-S_{y}+\left(32\times P\times x_{2}\right)/\left(\pi *{x_{1}}^{3}\right)\\ g_{2}\left(x\right)=-\Delta_{\max}+\left(64\times P\times {x_{2}}^{3}\right)/\left(3\times E\times \pi \times {x_{1}}^{4}\right) \end{array}\}$$

The related parameters are given as follows. The maximum allowed stress $S_{y}$ is selected as $300\times 10^{3}$ in kPa, the elastic modulus E is selected as $207\times 10^{6}$ in kPa, ρ is selected as 7800 in kg/m^3^, the load P is selected as 1 in kN, and the maximum allowed deflection $\Delta_{\max}$ is selected 0.005 in mm.

#### 4.2.6 Honeycomb heat sink design problem

Honeycomb assemblies have garnered widespread recognition across multiple engineering disciplines for their exceptional attributes and versatile functionality. These assemblies stand out in applications demanding efficient thermal regulation, absorption of impact energy, and support against structural loads. The unique combination of thermal properties and geometric configuration of honeycomb structures yields several key advantages [96]. Firstly, their significant surface-to-area ratio amplifies their utility in heat transfer applications, enabling superior efficiency in scenarios where the surface area is paramount. Secondly, the architectural layout of honeycomb structures promotes a streamlined fluid flow, presenting an optimal solution for scenarios requiring the preservation of fluid dynamics with reduced energy dissipation. Lastly, the construction materials, commonly including metals such as aluminium, possess high thermal conductivity, ensuring effective heat dispersion.

Central to heat sink optimization is the principle of thermal resistance, which measures the challenge heat encounters during transfer through materials. Optimizing a heat sink involves minimizing this resistance, thereby enabling quicker heat dissipation. Materials like aluminium are often chosen for their low thermal resistance, balancing efficiency with cost and weight considerations. The unique geometry of honeycomb structures significantly increases the surface area available for convective heat transfer, as indicated by the Nusselt number (Nu). This dimensionless metric assesses the convective heat transfer rate relative to conductive heat transfer, guiding the design process to maximize Nu through strategic modifications of the heat sink's geometry and surface characteristics. Fluid dynamics principles, particularly the friction factor (ff), are crucial in evaluating the resistance fluid experiences flowing through the heat sink. Optimizing design to reduce the friction factor ensures efficient fluid flow with minimal energy loss, which is crucial for systems where air or liquid serves as the cooling medium. The choice of material impacts not only the thermal conductivity of the heat sink but also its durability, weight, and overall cost. Aluminium is frequently selected for its excellent thermal conductivity and cost-effectiveness, highlighting the need for materials that enhance thermal performance without compromising structural integrity or economic viability. Adjusting the honeycomb structure's geometry, including cell size, shape, and arrangement, directly affects thermal performance. Design variables such as cross-sectional areas and dimensions of components are meticulously optimized to balance thermal efficiency with physical and mechanical constraints. Design optimization also accounts for the specific heat load and ambient conditions expected in operation. This ensures that the heat sink design is robust and capable of performing under varied and potentially extreme conditions.

In the background of heat sink and heat exchanger design, honeycomb structures, as elucidated in sources [92], demonstrate exceptional proficiency. Their structural design facilitates stellar heat transfer performance while simultaneously optimizing space utilization and curbing energy wastage. When crafting an exemplary heat sink, especially one adorned with hexagonal aluminium fins, the analysis of five decision parameters becomes imperative. These parameters, encompassing fin dimensions, inter-fin spacing, material gauge, and fin elevation, critically impact the heat sink's thermal and frictional performance. Evaluation of such a heat sink's efficiency hinges on analyzing two essential objective functions: the friction factor (ff) and the Nusselt number (Nu). The Nu, a dimensionless metric, gauges the effectiveness of convective over conductive heat transfer at the fluid boundary, with higher values indicating enhanced convective heat transfer efficiency. Conversely, the ff metric quantifies the frictional resistance encountered by the fluid navigating through the heat sink, underscoring the importance of minimizing friction to bolster fluid flow efficiency and minimize energy expenditure. Through meticulous optimization of these parameters, the aim is to engineer a heat sink design that optimally balances heat transfer enhancement against frictional resistance, thereby elevating the efficacy and efficiency of thermal management systems. The objective functions are detailed as follows.

(40) $$\begin{array}{c} \text{Maximize}:f_{1}\left(x\right)=-Nu\\ \text{Minimize}:f_{2}\left(x\right)=FF \end{array}\}$$

(41) $$FF=0.4753-0.0181\times h+0.0420\times t+5.481\times 10^{-3}\times S_{y}-0.0191\times \theta -3.416\times 10^{-6}\times Re-8.851\times 10^{-4}\times h\times S_{y}+8.702\times 10^{-4}\times h\times \theta +1.536\times 10^{-3}\times t\times \theta -2.761\times 10^{-6}\times t\times Re-4.400\times 10^{-4}\times S_{y}\times \theta +9.714\times 10^{-7}\times S_{y}\times Re+6.777\times 10^{-4}\times h^{2}$$

(42) $$Nu=89.027+0.300\times h-0.096\times t-1.124\times S_{y}-0.968\times \theta +4.148\times 10^{-3}\times Re+0.0464\times h\times t-0.0244\times h\times S_{y}+0.0159\times h\times \theta +4.151\times 10^{-5}\times h\times Re+0.1111\times t\times S_{y}-4.121\times 10^{-5}\times S_{y}\times Re+4.192\times 10^{-5}\times \theta \times Re$$

**Fig. 15:**
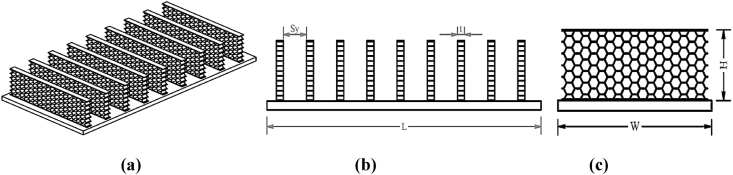
Honeycomb heat sink design: (a) Isometric view, (b) side view, (c) front view.

The bounds of the decision variables are given as follows. Decision vector $x={\left(h,t,S_{y},\theta ,Re\right)}^{T}$, 6≤t≤15 is the fin thickness in mm, $20\leq S_{y}\leq 40$ is the longitudinal pitch in mm, 20≤h≤60 is the fin height in mm, 0≤θ≤30 is the angle of attack in degrees, and 8000≤Re≤25000 is the Reynolds number. The assembly of the honeycomb heat sink is revealed in Fig. 15.

### 4.3 Results of real-world engineering problems

Table 9 within this study presents an in-depth statistical analysis comparing the MORCOA against a range of alternative optimization algorithms, with a particular focus on their efficacy in solving diverse engineering design problems. The primary metric employed for this evaluation is the GD, a measure that assesses the proximity of an algorithm's solutions to the true Pareto front, thereby indicating its convergence capability. The data compiled in Table 9 clearly demonstrates MORCOA's superior performance relative to the other considered algorithms across a variety of engineering design scenarios. This superiority is quantitatively underscored by MORCOA's consistently lower GD values across the board. Such lower GD values are indicative of MORCOA's superior convergence properties, showcasing its ability to closely approximate the true Pareto front more effectively than its counterparts in these complex engineering optimization tasks. In addition, Friedman's ranking test (FRT) values are also provided.

**Table 9:** All performance metrics for RW engineering problems.

		RW1	RW2	RW3	RW4	RW5	RW6	Average FRT
		Mean (STD)	Mean (STD)	Mean (STD)	Mean (STD)	Mean (STD)	Mean (STD)	
GD	MORCOA	11.7508 (0.9503)	0.0023 (0.0001)	8.3190 (1.7755)	0.0053 (0.0001)	0.0008 (0.0001)	0.0284 (0.0063)	**1.556**
	NSGA-II	12.5788 (0.7033)	0.0258 (0.0206)	10.7140 (1.3223)	0.0174 (0.0154)	0.0009 (0.0001)	0.0329 (0.0040)	2.667
	MOAGDE	14.8503 (2.4595)	0.0024 (0.0002)	13.7358 (0.0460)	0.0984 (0.1373)	0.0010 (0.0000)	0.0582 (0.0079)	3.500
	MOIPSO	14.2692 (2.8363)	0.0088 (0.0120)	15.0269 (0.7756)	0.0065 (0.0016)	0.0012 (0.0001)	0.1140 (0.0258)	3.556
	MOGBO	12.9851 (0.9057)	0.0032 (0.0004)	14.5481 (0.3374)	0.2188 (0.2520)	0.0009 (0.0000)	0.1175 (0.0511)	3.722
IGD	MORCOA	3.1989 (0.1817)	0.0804 (0.0006)	137.5811 (1.7455)	0.1689 (0.0057)	0.0116 (0.0005)	3.6749 (0.1278)	**1.611**
	NSGA-II	3.3183 (0.2034)	0.0860 (0.0093)	158.4183 (1.1874)	0.1814 (0.0040)	0.0122 (0.0000)	3.5254 (0.0401)	2.611
	MOAGDE	3.6782 (0.1181)	0.0795 (0.0015)	173.1956 (19.0006)	0.2905 (0.0245)	0.0122 (0.0002)	4.8227 (0.0564)	3.778
	MOIPSO	3.9072 (0.4449)	0.0821 (0.0053)	150.8403 (5.5809)	0.2125 (0.0182)	0.0140 (0.0023)	4.1550 (0.2708)	3.444
	MOGBO	4.0492 (0.0946)	0.0788 (0.0022)	145.5407 (1.1376)	0.2622 (0.0383)	0.0137 (0.0008)	4.2040 (0.1945)	3.556
HV	MORCOA	0.4308 (0.0288)	0.7619 (0.0002)	0.2644 (0.0001)	0.8377 (0.0006)	0.7625 (0.0001)	0.6856 (0.0016)	**1.778**
	NSGA-II	0.3980 (0.0317)	0.7631 (0.0003)	0.2608 (0.0001)	0.8294 (0.0040)	0.7622 (0.0003)	0.6813 (0.0006)	2.889
	MOAGDE	0.5229 (0.0491)	0.7618 (0.0004)	0.2597 (0.0019)	0.8370 (0.0014)	0.7624 (0.0002)	0.6730 (0.0010)	3.444
	MOIPSO	0.5349 (0.1040)	0.7633 (0.0001)	0.2623 (0.0007)	0.8360 (0.0011)	0.7619 (0.0004)	0.6810 (0.0024)	3.500
	MOGBO	0.5234 (0.0519)	0.7613 (0.0000)	0.2626 (0.0006)	0.8347 (0.0019)	0.7620 (0.0001)	0.6798 (0.0024)	3.389
SD	MORCOA	0.4308 (0.0288)	0.6330 (0.0246)	0.4374 (0.0192)	0.4587 (0.0839)	0.7325 (0.0238)	0.3059 (0.0380)	**1.556**
	NSGA-II	0.3980 (0.0317)	0.6486 (0.0162)	0.4573 (0.0693)	0.4786 (0.0660)	0.7359 (0.0313)	0.3522 (0.0826)	2.167
	MOAGDE	0.5229 (0.0491)	0.6325 (0.0254)	1.1451 (0.1115)	1.0652 (0.1027)	0.7352 (0.0385)	0.7581 (0.0866)	3.722
	MOIPSO	0.5349 (0.1040)	0.6309 (0.0777)	0.8469 (0.0499)	0.6856 (0.0746)	0.7743 (0.0393)	0.4975 (0.0821)	3.833
	MOGBO	0.5234 (0.0519)	0.6320 (0.0887)	0.8123 (0.0405)	0.9726 (0.2967)	0.8052 (0.0390)	0.4743 (0.0232)	3.722
SP	MORCOA	5.9813 (0.2132)	0.1509 (0.0041)	16.7934 (2.5553)	0.2317 (0.0433)	0.0272 (0.0011)	4.0412 (0.3887)	**2.111**
	NSGA-II	5.4603 (0.4928)	0.1781 (0.0363)	18.9633 (5.0194)	0.2974 (0.0742)	0.0288 (0.0013)	4.4676 (0.5574)	3.611
	MOAGDE	6.5811 (0.6590)	0.1564 (0.0049)	32.3347 (3.2767)	0.4232 (0.0453)	0.0280 (0.0018)	6.4572 (0.4154)	3.722
	MOIPSO	7.2073 (1.8840)	0.2347 (0.0724)	33.1047 (3.6446)	0.3181 (0.0297)	0.0292 (0.0004)	5.7491 (0.6891)	3.167
	MOGBO	6.7141 (0.6702)	0.1514 (0.0188)	29.7615 (3.2233)	1.7264 (1.9322)	0.0297 (0.0014)	4.9114 (0.1068)	2.389

In this section, Table 9 provides a detailed statistical benchmark, offering a comparative analysis of MORCOA and other optimization algorithms in tackling various engineering design challenges. The primary metric for this evaluation is the GD, which measures how closely an algorithm's generated solutions approach the true Pareto front, indicating the algorithm's convergence efficiency. The data in Table 9 shows that MORCOA achieves consistently lower GD values, highlighting its superior convergence capabilities and proficiency in more accurately approximating the true Pareto front within complex engineering optimization landscapes. Additionally, Table 9 evaluates the algorithms using the IGD metric, which assesses how effectively these algorithms solve a series of engineering design problems. The analysis reveals that MORCOA consistently outperforms other prominent algorithms such as NSGA-II, MOIPSO, MOGBO, and MOAGDE across five distinct real-world engineering challenges. This performance level underscores MORCOA's competitive advantage and robustness, especially in contexts where performance margins among different algorithms are relatively narrow. Table 9 further broadens the scope of our analysis by incorporating additional pivotal performance indicators: SD, SP, and HV, each offering unique insights into the efficacy of the optimization algorithms under consideration:

The comparative results reveal a notable trend: MORCOA, the algorithm introduced in this study, exhibits superior performance in the majority of cases when evaluated against the backdrop of these metrics. Specifically, MORCOA outperforms its competitors in five out of six scenarios for the Spread metric. This consistent superiority underlines MORCOA's exceptional capability not only to discover a broad spectrum of optimal solutions but also to ensure that these solutions are evenly distributed across the entirety of the Pareto front. MORCOA also shows outstanding performance in the Spacing metric, ensuring an equitable distribution of solutions across the Pareto front, which is critical in multi-objective optimization to avoid solution clustering and guarantee comprehensive exploration of the solution space. In most of the scenarios assessed, MORCOA surpasses its competitors, further highlighting its efficiency in navigating multi-objective optimization challenges. The extensive statistical analysis provided across these tables illuminates the durability and adaptability of MORCOA in managing a variety of complex engineering design problems, demonstrating superior performance over other established algorithms across different evaluative metrics. This detailed analysis showcases MORCOA's dominance in striking an optimal balance between finding a comprehensive set of solutions and ensuring their equitable distribution, thereby enhancing the overall decision-making process in multi-objective optimization scenarios.

**Fig. 16:**
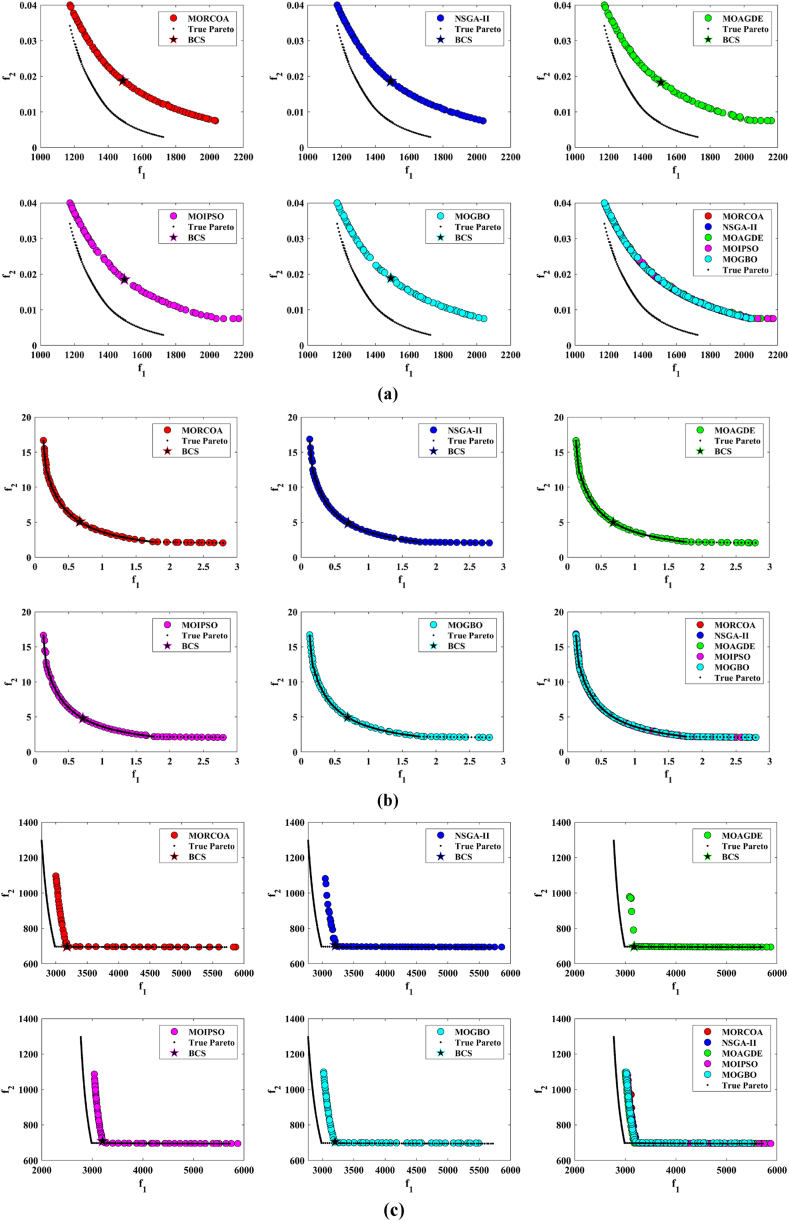

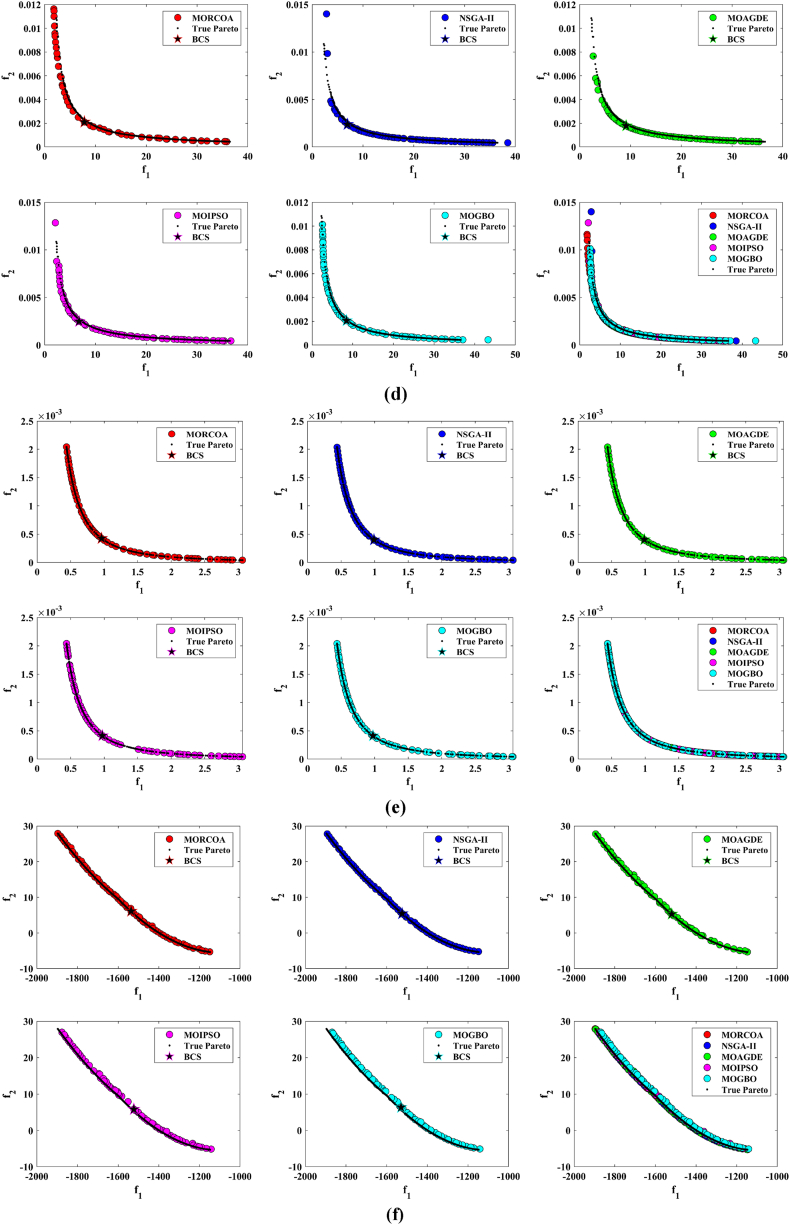
PF obtained by all algorithms for RW problems; (a) RW1, (b) RW2, (c) RW3, (d) RW4, (e) RW5, (f) RW7

To definitively demonstrate that the Pareto fronts generated by MORCOA are more aligned with the optimal solutions than those produced by alternative algorithms, this study includes detailed visual evidence in Fig. 16. This illustration compares the true Pareto fronts with those derived through various algorithms for the engineering design challenges addressed. A meticulous examination of these visuals reveals several critical insights into MORCOA's performance efficacy. Specifically, Fig. 16 clearly illustrates that the Pareto fronts realized by MORCOA more closely approximate the true Pareto fronts than those generated by other algorithms, signalling a higher precision in MORCOA's solution set. This proximity to the true Pareto fronts evidences MORCOA's superior capability in identifying solutions that adeptly balance the inherent trade-offs among the multiple objectives characteristic of these engineering tasks.

MORCOA's ability to produce well-distributed solution sets across the objective space stands out distinctly. Contrary to other algorithms, which may exhibit solution clustering or significant gaps, MORCOA spans a more extensive portion of the objective space, reflecting a deep and varied exploration of possible solutions. This broad distribution is imperative for encapsulating a comprehensive array of trade-offs, thus offering decision-makers a wide selection of optimal choices. The consistent achievement of MORCOA across diverse engineering design challenges, as depicted in Fig. 16, affirms its robustness and versatility. Regardless of the complexity of the structural or mechanical system in question, MORCOA invariably yields Pareto fronts that are markedly congruent with the true fronts. The graphical presentations in Fig. 16 provide a vivid and intuitive validation of MORCOA's operational effectiveness.

**Fig. 17:**
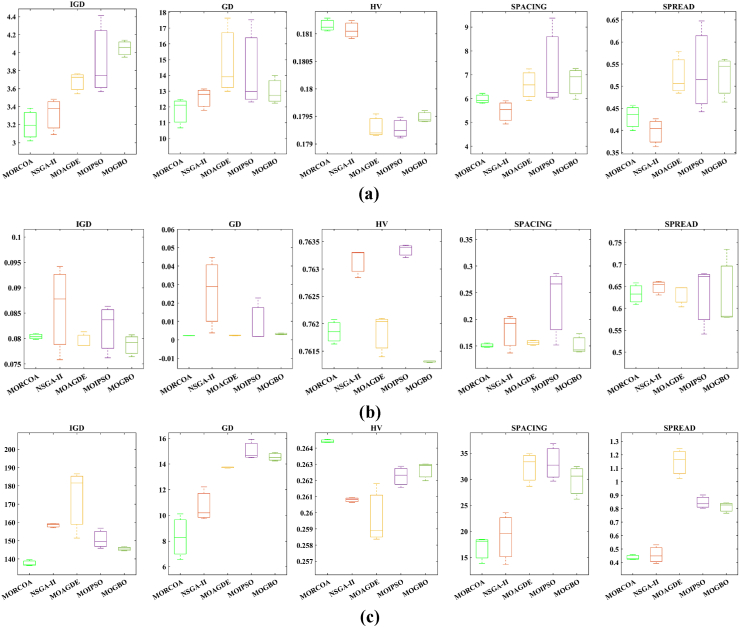

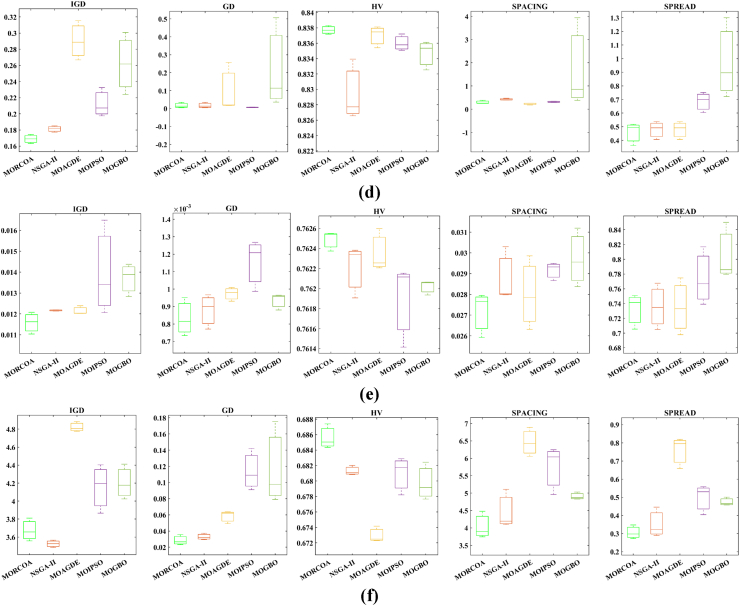
Boxplots obtained for all algorithms for RW problems: (a) RW1, (b) RW2, (c) RW3, (d) RW4, (e) RW5, (f) RW6

In this comprehensive study, the inclusion of box plots, as shown in Fig. 17, for all metrics across the entire set of problems provides a detailed statistical overview, further substantiating the reliability and competency of the MORCOA when compared with state-of-the-art algorithms. These box plots serve as a visual testament to MORCOA's performance, offering insights into the central tendency, dispersion, and outliers of the results obtained for each metric, thereby facilitating a granular analysis of its effectiveness across various engineering design challenges. The box plots meticulously detail the performance distribution for metrics such as GD, IGD, Spread, Spacing, and HV. Through these plots, MORCOA's ability to consistently achieve lower GD and IGD values is visually highlighted, demonstrating its superior convergence towards the true Pareto fronts. Similarly, the Spread and Spacing metrics depicted in the box plots reveal MORCOA's effectiveness in ensuring a diverse yet evenly distributed set of solutions across the Pareto front, an essential aspect of multi-objective optimization that underscores the algorithm's ability to explore the solution space comprehensively. Moreover, the HV metric, as visualized through the box plots, underscores MORCOA's capacity to cover a significant volume of the objective space, indicating not only the quality but also the quantity of the solutions it generates. This extensive coverage affirms MORCOA's adeptness in identifying a broad spectrum of optimal solutions, thereby providing decision-makers with a wide range of viable options to consider.

**Fig. 18:**
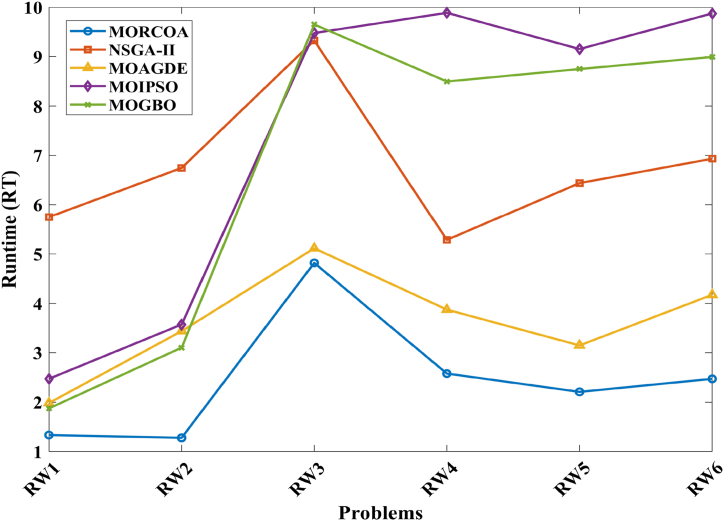
RT analysis of all algorithms for real-world problems.

Runtime (RT) analysis is crucial for comparing the efficiency of the proposed algorithm, assessing its scalability with problem complexity, and ensuring practical applicability in real-time scenarios. To better understand the performance of all algorithms, Table 10 and Fig. 18 are provided to visualize their runtimes across the different real-world problems.

**Table 10:** Average RT values of all algorithms after 30 individual runs.

Problem	MORCOA	NSGA-II	MOAGDE	MOIPSO	MOGBO
RW1	1.33458	5.7495	1.9835	2.4741	1.8739
RW2	1.2784	6.7453	3.4391	3.5737	3.1047
RW3	4.8194	9.3278	5.1145	9.4795	9.6519
RW4	2.5792	5.2848	3.8746	9.8874	8.4963
RW5	2.2089	6.4379	3.1499	9.1548	8.7496
RW6	2.4718	6.9318	4.1745	9.8741	8.9935

It is observed from Fig. 18 and Table 10 that MORCOA generally exhibits the lowest runtime across most problems, and it shows significant efficiency, particularly in RW1 and RW2. NSGA-II consistently has higher runtimes compared to other algorithms and demonstrates less efficiency, particularly in RW2 and RW3. MOAGDE's runtimes are generally in the middle range, higher than MORCOA but lower than NSGA-II for most problems and exhibit balanced performance across all problems. MOIPSO exhibits high runtimes, especially in RW3, RW4, RW5, and RW6 and indicates less efficiency in handling these specific problems. MOGBO is similar to MOIPSO, shows higher runtimes in later problems and demonstrates a trend of increased runtime in more complex problems. The RT analysis highlights the efficiency of MORCOA in comparison to other algorithms. MORCOA consistently shows lower runtimes, indicating its suitability for real-world problems where computational efficiency is crucial.

The aggregate analysis provided by box plots and RT analysis, across all considered metrics and problems, provides a depiction of MORCOA's reliability and superior performance. Compared to other contemporary algorithms, MORCOA demonstrates enhanced efficiency, robustness, and adaptability in addressing the multifaceted requirements of complex engineering design problems. The comprehensive visual and statistical evidence laid out in the box plots and RT analysis clearly supports the statement that MORCOA stands as a more competent solution in the landscape of multi-objective optimization algorithms, offering a reliable and effective tool for attempting the complicated trade-offs and challenges inherent in engineering optimization tasks.

### 4.4 Limitations of this study

In Table 6, Table 7, the performance of MORCOA in terms of Spacing and Spread metrics shows some variability, especially when compared to state-of-the-art algorithms. These limitations can be attributed to several factors.

• While effective in maintaining diversity, DEBCD may sometimes result in suboptimal spacing. This occurs because the elimination process might remove solutions that could have contributed to a more even distribution.
• The ZDT and DTLZ test suites encompass a wide range of problem complexities, including deceptive Pareto fronts and varying degrees of difficulty. MORCOA's transient response behaviour is highly effective for certain problem types but may struggle with others, leading to variations in spacing and spread performance.
• Achieving this balance perfectly across all test problems is challenging. In some cases, the algorithm might over-exploit certain regions, leading to clustered solutions and poor spacing.
• Although MORCOA is designed to handle a range of optimization problems, its efficiency and effectiveness may diminish with increasing problem scale and dimensionality. Large-scale problems require more computational resources and sophisticated handling of multi-dimensional spaces, where MORCOA might struggle to maintain its performance.

To enhance the robustness and efficiency of MORCOA, the following strategies could be implemented: (i) Introducing mechanisms that dynamically adjust the resistance and capacitance parameters during the optimization process based on real-time feedback can improve the algorithm's adaptability to different problem complexities; (ii) Incorporating advanced diversity preservation techniques, such as clustering-based methods or niche-preserving strategies, can help maintain a well-distributed Pareto front and improve spacing metrics; (iii) Combining MORCOA with other optimization techniques that excel in different areas of MOOPs, such as differential evolution or particle swarm optimization, could leverage the strengths of multiple algorithms, leading to better overall performance; (iv) Developing more efficient algorithms and parallel processing techniques can help MORCOA scale better with increasing problem size and complexity, making it more applicable to large-scale optimization problems.

While MORCOA shows promise in addressing multi-objective optimization challenges, acknowledging and addressing its limitations is crucial for further development. By implementing adaptive parameter tuning, enhancing diversity preservation, exploring hybrid approaches, and improving scalability, MORCOA's performance can be significantly enhanced, making it a more robust and versatile tool for complex optimization tasks.

## 5 Conclusions and future scopes

This investigation has rigorously validated the efficacy of the proposed MORCOA through its application to a series of numerical benchmarks and real-world engineering challenges, including the complex honeycomb heat sink design problems. Across these various testing scenarios, MORCOA has consistently demonstrated a superior performance profile when benchmarked against state-of-the-art MOO algorithms. Key findings from this study include MORCOA's exceptional ability to converge towards the true Pareto fronts, significantly outperforming competitors such as NSGA-II, MOIPSO, MOGBO, and MOAGDE. This was particularly evident in engineering design problems where MORCOA not only identified different optimal solutions but also ensured these solutions were evenly distributed across the Pareto front, showcasing its robust capability in achieving a comprehensive exploration of the solution space. In the context of the honeycomb heat sink design optimization, MORCOA's superior performance was highlighted by its proficient handling of the design's complex thermal and structural requirements. By effectively balancing objectives like minimizing thermal resistance and optimizing fluid flow, MORCOA illustrated its adaptability and precision in solving the complicated design spaces typical of real-world engineering problems. The statistical analysis, enriched by the use of performance metrics such as GD, IGD, Spread, Spacing, and HV, provided a quantitative foundation for MORCOA's evaluation. The algorithm's dominance in these metrics across various problem sets reinforces its status as a highly reliable and efficient tool for multi-objective optimization tasks. The study's comprehensive approach, combining theoretical insights with empirical analysis, has clearly established MORCOA as a valuable asset in the field of optimization. Its proven capability to deliver high-quality, diverse, and evenly distributed solutions across a wide range of optimization problems positions MORCOA as a leading choice for engineers and researchers facing complex multi-objective design and optimization challenges.

Given the promising results obtained in this study, several avenues for future research emerge: (i) While MORCOA has demonstrated robust performance, exploring potential algorithmic enhancements could further improve its efficiency. This might include integrating adaptive mechanisms for parameter tuning or exploring hybridization with other optimization techniques to enhance its exploratory and exploitative capabilities; (ii) Extending the application of MORCOA to other complex, real-world problems beyond engineering design could provide insights into its adaptability and efficiency in different domains, such as biomedical, financial, and environmental engineering; (iii) Investigating the scalability of MORCOA, particularly in handling problems with a higher number of objectives and decision variables, would be valuable; (iv) As new optimization algorithms are developed, conducting comparative studies remains crucial. Future research should continue to benchmark MORCOA against emerging algorithms to ensure its competitiveness and identify areas for improvement; (v) Developing decision support systems incorporating MORCOA's optimization capabilities could be beneficial.

## Funding

For the research included in this submission, the authors received no organizational or financial support.

## Data availability statement

Upon reasonable request, the corresponding author provides access to the datasets used in this study.

## Ethical approval

There is no need for ethical approval, according to the authors.

## CRediT authorship contribution statement

**Sowmya Ravichandran:** Writing – original draft, Visualization, Validation, Software, Methodology, Data curation, Conceptualization. **Premkumar Manoharan:** Writing – original draft, Validation, Supervision, Software, Resources, Methodology, Investigation, Data curation, Conceptualization, Mohammad Sh. Daoud, Writing – review & editing, Visualization, Formal analysis. **Deepak Kumar Sinha:** Writing – review & editing, Software, Methodology, Formal analysis, Data curation. **Pradeep Jangir:** Writing – review & editing, Visualization, Software, Formal analysis. **Laith Abualigah:** Writing – review & editing, Visualization, Formal analysis. **Thamer A.H. Alghamdi:** Writing – review & editing, Visualization, Formal analysis.

## Declaration of competing interest

The authors declare that they have no known competing financial interests or personal relationships that could have appeared to influence the work reported in this paper.

## Figures and Tables

**Table A1:** Control parameters of all selected algorithms

Algorithm	Parameter	Value
MOIPSO	Population size	100
	Cognitive coefficient (c_1_) and social coefficient (c_2_)	1.5
	$w_{\max}$ and $w_{\min}$	0.9 and 0.4
	Maximum number of generations	30000
NSGA-II	Population size	100
	Crossover probability	0.9
	Mutation probability	0.3
	Distribution index for crossover and mutation	20
	sep	0.1
	Maximum number of generations	30000
MOAGDE	Population size	100
	Crossover probability	0.9
	Differential weight	0.5
	Maximum number of generations	30000
	Adaptive parameter adjustment	Adaptive over iterations
MOGBO	Population size	100
	pr	0.5
	Maximum number of generations	30000
MORCOA (proposed)	Population size	100
	t and τ	Adaptive adjustments
	Maximum number of generations	30000
